# Ghost-in-the-Machine reveals human social signals for human–robot interaction

**DOI:** 10.3389/fpsyg.2015.01641

**Published:** 2015-11-04

**Authors:** Sebastian Loth, Katharina Jettka, Manuel Giuliani, Jan P. de Ruiter

**Affiliations:** ^1^Psycholinguistics, Faculty of Linguistics and Literary Studies, Bielefeld UniversityBielefeld, Germany; ^2^Center for Human-Computer Interaction, Department of Computer Sciences, University of SalzburgSalzburg, Austria

**Keywords:** human–robot interaction, social behavior, eye tracking, interaction strategies, social signals, intention recognition

## Abstract

We used a new method called “Ghost-in-the-Machine” (GiM) to investigate social interactions with a robotic bartender taking orders for drinks and serving them. Using the GiM paradigm allowed us to identify how human participants recognize the intentions of customers on the basis of the output of the robotic recognizers. Specifically, we measured which recognizer modalities (e.g., speech, the distance to the bar) were relevant at different stages of the interaction. This provided insights into human social behavior necessary for the development of socially competent robots. When initiating the drink-order interaction, the most important recognizers were those based on computer vision. When drink orders were being placed, however, the most important information source was the speech recognition. Interestingly, the participants used only a subset of the available information, focussing only on a few relevant recognizers while ignoring others. This reduced the risk of acting on erroneous sensor data and enabled them to complete service interactions more swiftly than a robot using all available sensor data. We also investigated socially appropriate response strategies. In their responses, the participants preferred to use the same modality as the customer’s requests, e.g., they tended to respond verbally to verbal requests. Also, they added redundancy to their responses, for instance by using echo questions. We argue that incorporating the social strategies discovered with the GiM paradigm in multimodal grammars of human–robot interactions improves the robustness and the ease-of-use of these interactions, and therefore provides a smoother user experience.

## Introduction

**FIGURE 1:**
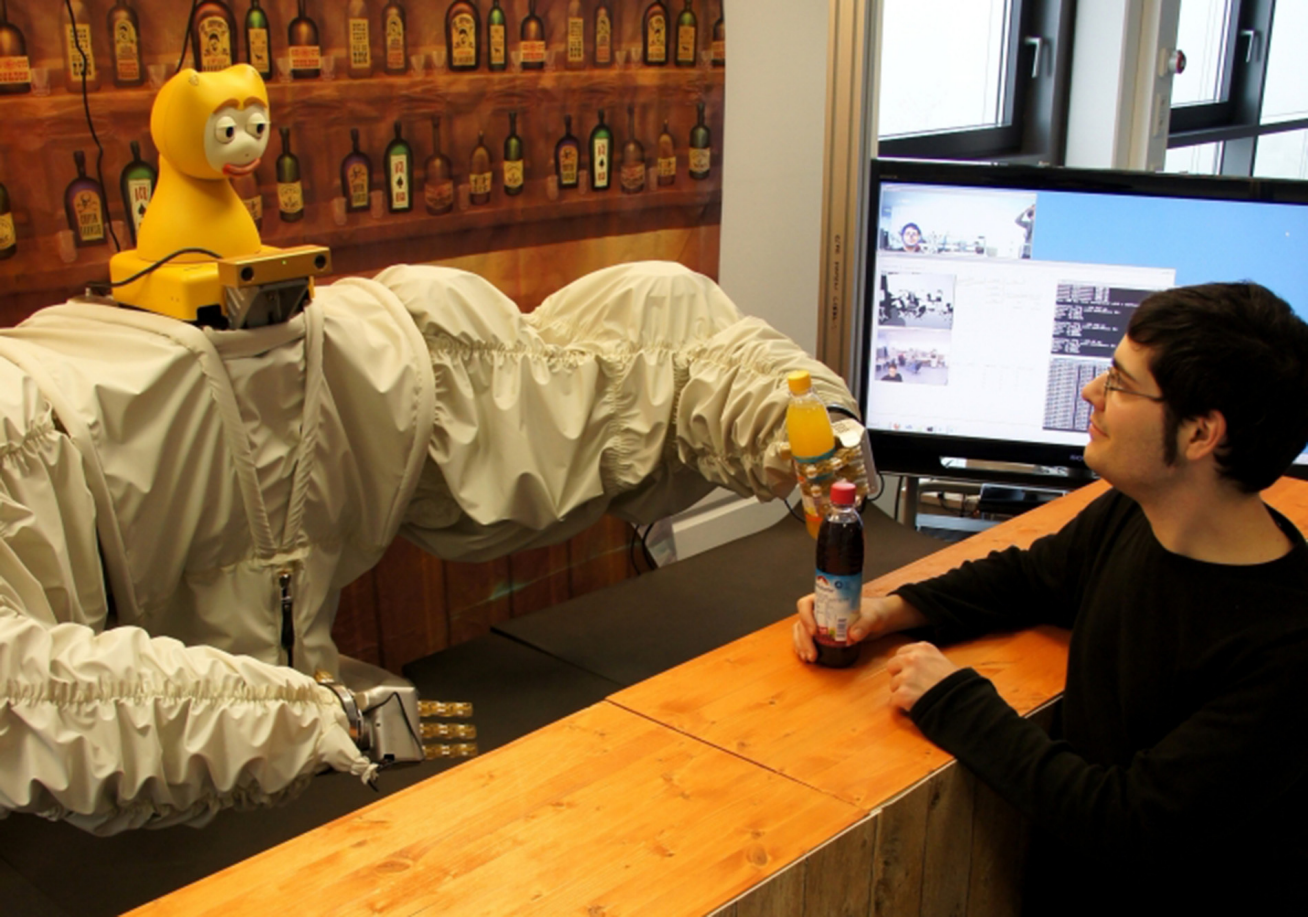
**Robotic bartender JAMES serving drinks to a customer**.

Robotic agents are increasingly used for interacting with humans in public spaces, e.g., for providing information as a museum guide (Yousuf et al., 2012) or serving snacks (Lee et al., 2009). We used the bar scenario as challenging example for a social environment. The robot acts as bartender that accepts drink orders from human customers and serves drinks (see **Figure 1**). Thus, the robot has to complete the task (i.e., serving the correct drink) and, importantly, it has to understand and produce socially acceptable behavior. The bartending robot is located at a fixed position behind the bar. Typically multiple customers are in close proximity in front of the bar. First, the robot has to identify the customers who would like to initiate an interaction (Loth et al., 2013). Once the interaction has been established, the robot has to sense the customer’s dialog moves, reason about them and produce an appropriate response (Petrick and Foster, 2012). That means that the robot has to have an understanding of the user’s engagement behavior (Sidner and Lee, 2003; Sidner et al., 2005), recognize the user’s intentions (Gray et al., 2005), and produce socially appropriate responses (Petrick and Foster, 2012; Breazeal et al., 2013). Thus, reliable, robust, and social interaction policies are crucial for enabling users to interact intuitively with a robot (Goodrich and Schultz, 2007). Additionally, users enjoy interacting with a social robot to a greater extent than with a purely task-oriented system (Foster et al., 2012; Giuliani et al., 2013). In order to develop empirically driven and socially appropriate interaction policies for the robotic bartender, we tested (a) whether the recognizer data are sufficient for entertaining a socially credible interaction, (b) which recognizer modality was the most informative at each stage of the interaction, and (c) what kind of repair strategies humans employ in a social interaction. We used the Ghost-in-the-Machine paradigm (GiM; Loth et al., 2014) because the results can be transferred directly into robot policies as the human participants are presented the same recognizer data as the robotic planner.

## Related Work

Human–human interaction is highly fluent and can be regarded as the gold standard for human–robot interaction. Thus, we briefly review the mechanisms involved in human–human interaction and how they can be modeled in a robotic agent. Typically empirical studies were designed for investigating particular aspects of robotic interaction policies. We review previous studies with respect to how transferable their results are. In particular, we focus on whether the data that the human participants observed in the study were comparable to the kind of data that the robotic planner has access to. We highlight potential problems in these studies before describing our GiM study in more detail.

### Social Signals

Interacting with other humans is perceived as most natural and intuitive compared to robotic or virtual agents. Thus, in order to improve the interaction with the robotic bartender, we have to understand how humans communicate their intentions in a social environment. Levinson (1995) argued that humans recognize the intentions of others from communicative actions. These are composed of one or more observable, basic actions in several modalities (e.g., Levinson, 1995; Vinciarelli et al., 2012). We refer to these observable actions as *social signals*. These basic actions are the starting point for human and robotic recognition. Humans identify basic actions such as walking by matching the percept against a representation in memory (Jeannerod, 2006). But it is not clear how humans understand the intention of somebody who is walking (Levinson, 1995). In robots, sensor data are typically categorized by trained classifiers into one type of action. For example, the computer vision recognizes dynamic actions such as waving, walking, and running (for review, see Poppe, 2010). Also, the user’s pose (Shotton et al., 2013), hands and faces can be tracked (Baltzakis et al., 2012; Gaschler et al., 2012) for identifying deictic gestures in (close to) real-time (Pateraki et al., 2014). The automatic speech recognition (ASR) aims to recognize the user’s utterance by matching it against a dictionary (or a grammar and a dictionary). In general, recognizers transform a constant stream of data from the sensors (e.g., microphone, camera) into distinct events such as an instance of waving or a specific speech utterance. However, robotic recognizers generally require a substantial amount of computation. Additionally, a dimly lit and noisy bar location challenges them such that their results tend to be more error-prone. Human bartenders face a similar problem as they cannot constantly monitor each potential customer in a busy place given that their cognitive resources are limited (e.g., Broadbent, 1969). This holds especially for monitoring within a single sensory modality^1^ (Allport et al., 1972; Mcleod, 1977). Thus, the human bartenders have to employ heuristics, for instance by focussing on distinctive aspects of the scene (e.g., the distance of customers to the bar).

Humans select the relevant aspects by relying on prior shared knowledge about the expected behavior and signals of both partners in the interaction (Levinson, 1995). These expectations also determine the attentional focus of the partners. Once the signal is identified, humans evaluate plausible intentions, i.e., the human recipient tries to attribute a plausible social intention to the signal (Grice, 1957; Levinson, 1995). This is essential as it makes an action meaningful. But correctly identifying social signals and understanding other’s intentions is logically intractable and thus, prior shared knowledge and heuristics are required (Levinson, 1995). For a robotic agent, this knowledge has to be explicated and formalized, e.g., in scripts that capture the conventionalized sequence of events (Schank and Abelson, 1977; Abelson, 1981) or the computational AIRBUS model that combines prior expectations, knowledge about conventions, and recognized signals during interactions (De Ruiter and Cummins, 2012). By explicating this implicit social knowledge, we can improve the robustness and the perceived quality of human–robot interaction. At the same time, the computational efforts can be limited to extracting only the necessary information by identifying the relevant recognizer modalities. For example, in the bar scenario customers signal to a member of staff that they would like to place an order by positioning themselves very close to the bar and turning toward the counter or a member of staff (Loth et al., 2013). Thus, only these two modalities have to be attended in order to identify new customers reliably. Furthermore, the participants only attended the body posture of potential customers if they were close to the bar whereas the body posture was irrelevant for customers who were further away. This reduces the cognitive load of understanding the scene for a human observer even further. Using this hierarchical rule-set is also advantageous to the robot. By analysing the body posture of customers who are close to the bar only, the line of sight is less likely to be obstructed by objects and other customers and thus, the recognizer works more reliably with less computational efforts. Additionally, the robotic recognizers are subject to noise. By reducing the number of noisy data sources, the amount of potentially misleading recognizer data is also reduced. Thus, our central aim is to provide an empirical method for reliably identifying social signals and the relevant recognizer modalities they are signaled in.

Our review of human social cognition suggests that using prior knowledge and focussing on particular aspects of the scene (recognizer modalities) can reduce errors and computational efforts. However, this is achieved by ignoring substantial amounts of data which may sound counter-intuitive. But this is a general finding in human cognition. Humans focus on task-relevant aspects of the scene and ignore other events in the visual (inattentional blindness; Mack and Rock, 1998) and auditory domain (inattentional deafness; Dalton and Fraenkel, 2012). For example, Simons and Chabris (1999) asked their participants to count the number of passes played by a basketball team and argued that the frequent failures to notice a man in a gorilla costume who walked through the scene were due his irrelevance to the task. Thus, by selecting the aspects of the scene (recognizer modalities) appropriately, the robot’s performance becomes more human-like and more predictable to its human users. In turn, we aimed to identify which aspect of the scene is relevant before and during an interaction at the bar.

In a social interaction, producing socially acceptable behavior is equally important as understanding it. For example, in a task requiring users to sort blocks that were handed to them by a robot, they sorted the blocks on their own strategy, e.g., by color. Only if a short delay was included between stretching the robot’s arm and releasing the block, the users attended the robot’s gaze and used it as a sorting instruction (Admoni et al., 2014). Thus, the delay formed a social signal to attend the robot’s gaze direction. Also, users smile more often if the robot smiles at them (Krämer et al., 2013). In general, interacting with a robot that acts socially appropriately is perceived as more pleasing than with a purely task-oriented robot (Giuliani et al., 2013). Thus, we aim to identify social signals to be displayed by robotic bartenders that can be reliably interpreted by its customers.

### Methods of Deriving Interaction Models

Interaction models can be hand-crafted but are often partly based on empirical data. For example, hand-crafted models are typically adapted after an initial testing period in the wild such that the first model serves as test and data collection device. Other methods of gathering empirical data are computer games and the Wizard-of-Oz paradigm (WOz). In this review, we focus on how the relevant recognizer modalities were identified.

For detecting whether visitors intended to interact with a robotic receptionist, Michalowski et al. (2006) based their interaction model on proxemics (Hall, 1969). This hand-crafted model triggered a greeting as soon as a potential user was sufficiently close to the robot. But passers-by who accidentally came close to the robot felt disturbed when the robot greeted them out of the blue (Michalowski et al., 2006, p. 766). Thus, Rich et al. (2010) and Holroyd et al. (2011) used several multimodal cues that were partly inspired by research on human–human interaction (Schegloff and Sacks, 1973), e.g., the point of gaze. This is a highly informative cue but it can be difficult to measure in the wild. Importantly, it might not be accessible to humans in a busy environment and thus, not be part of the conventionalized social signals that we aim to identify. For example, in the bar setting less fine grained aspects of the scene such as the distance to the bar and the body or head orientation were most relevant (Loth et al., 2013). An initially hand-crafted interaction model can also be adapted to the user behavior during a test period of real-world interactions. For example, Bohus and Horvitz (2009a,b,c,d, 2010, 2011) implemented a number of sensors and recognizers in their static receptionist and trivia quiz platform, and more recently in a direction-giving robot (Bohus et al., 2014). They refined their engagement models constantly but they could not accommodate all user behavior (Bohus and Horvitz, 2009a). In particular, multiple users formed a challenge for these accounts (Michalowski et al., 2006; Bohus and Horvitz, 2009c) whereas our bar scenario typically involves multiple customers. Goodrich and Schultz (2007) classified these accounts as *proof-of-concept* because the users interacted with a given system and adapted their behavior. This was illustrated by the graphically simple WAITER game (Xu et al., 2010). Even though the manager participant had only indirect evidence, this participant adapted quickly to the abilities of the waiter participant that were manipulated by the game engine. This suggests that proof-of-concept approaches do not investigate what is intuitive to the users but how well they adapt to a given system. However, identifying the underlying psychological principles of natural behavior and designing the robot’s policies around them is more useful (Goodrich and Schultz, 2007).

Games with a purpose (GWAP; von Ahn and Dabbish, 2008) and in particular online games allow acquiring large data sets, e.g., as training data for machine learning accounts. In The-Restaurant-Game, users could engage online as waitress or customer (Orkin and Roy, 2007, 2009). Orkin and Roy (2009) derived a sequential graph of actions that was argued to reflect collective intelligence. After training the virtual agents on these data, they worked reasonably but also produced some errors, e.g., asking for selecting a starter after starters had just been served. Even though the players had an intentional structure in mind, this method did not capture this structure from the surface behavior (Orkin and Roy, 2009, p. 392).

The WOz paradigm is typically used for investigating the user behavior while s/he believes to interact with a real robot. But in fact, an informed assistant or another participant acts as a ‘wizard’ that controls the robot (Kelley, 1984; Fraser and Gilbert, 1991; Dahlbäck et al., 1993). For maintaining the illusion of a real robot and providing swift responses, the workload of controlling the robot sometimes has to be divided between several wizards which may cause inconsistencies in the robot’s behavior (Green et al., 2004; Rieser and Lemon, 2009). Several WOz studies also investigated the behavior of a single wizard. For example, for investigating when wizards asked for clarifications (Rieser and Lemon, 2009) and which mode of information presentation they selected (Rieser et al., 2011). In these studies, the distortion of an ASR was simulated by a typist translating the user’s speech into text and deleting or replacing words. However, in more than 80% of WOz studies, the wizards had access to immediate, unfiltered video and audio data of their users (Riek, 2012). In contrast, the robotic planner has to rely on the robot’s recognizers introducing delays, losses, and misinterpretations of data. This difference can impair the transferability of the findings into robotic decision policies. For example, Lee et al. (2009) collected WOz data and designed a script for their Snackbot. But the real-life evaluation showed that half of their script phrases were unsuitable (Lee et al., 2009, p. 11). Thus, it is important to ensure that the wizards and the robotic planner operate on the same type of data. For example, semantically analyzed data of the ASR component was presented to the wizards of a restaurant information system (Liu et al., 2009). This method is similar to our GiM approach (Loth et al., 2014) and we expand on this in our study.

Lichtenthäler et al. (2013) introduced the *Inversed Oz of Wizard* for investigating how a wizard would avoid a collision between a confederate and the robot under her/his control. In this setting, the wizard observed the confederate and the robot from the same room. Thus, the human observer could have subconsciously interpreted subtle cues in the motion patterns of the confederate that the robotic recognizers are not able to interpret reliably, e.g., by observing the motion preceding an attack in volleyball (Schorer et al., 2013) or a penalty kick in football (Noël et al., 2014), athletes can anticipate the actions of their opponents (also see Abernethy et al., 2007). This is more pronounced in everyday behaviors of groups as they tend to synchronize by subtly communicating their next movements to each other (Néda et al., 2000; Richardson et al., 2007; Lakens and Stel, 2011). Thus, especially in settings with multiple users such as the bar scenario, the robotic planner would not have access to the information that was essential to the human performance. In order to avoid this missing link, we carefully designed the GiM interface such that the human participant has access to the same information as the robotic planner.

## Materials and Methods

We aimed to (a) identify the social signals and relevant recognizer modalities in the bar scenario, (b) learn how the robotic bartender should respond to its customers in a socially appropriate way, and (c) combine these insights for developing strategies for recovering from false or inconclusive recognizer data that are socially acceptable and, specifically, less annoying to the customer than repeatedly asking for clarifications. Thus, we used the GiM paradigm (Loth et al., 2014). In this paradigm, the main participant (*ghost*) observes the scene through the eyes and ears of the robot, i.e., the ghost has access to the recognizer data but no direct video or audio link to the customers. Hence, the ghost and the robotic planner use the same data. In order to interact with the customers, the ghost has to select actions from the robot’s repertoire. In contrast to the typical WOz studies that focus on the user’s behavior, we are primarily interested in the behavior of the ghosts. For assessing the reliability of this paradigm, we compared our findings to earlier empirical studies that relied on real world observations (Brouwer et al., 1979) and experiments using natural stimuli (Loth et al., 2013, 2015).

In order to avoid confusion, we refer to the main participants as *ghosts* and to the participants who ordered drinks as *customers*.

### Participants

Thirty-one participants were recruited as main participants from the departmental participant pool (formed of linguistics and other students as well as university staff) in Bielefeld, Germany. They received aaa5 and a chocolate bar in exchange for their time and effort. The eye tracker could not be calibrated with two participants and their data were not included in the results.

The experiment and all procedures were approved by University Bielefeld’s Ethics Committee (EUB) under approval No4807. An informed written consent was collected prior to the experiment.

### Apparatus

**FIGURE 2:**
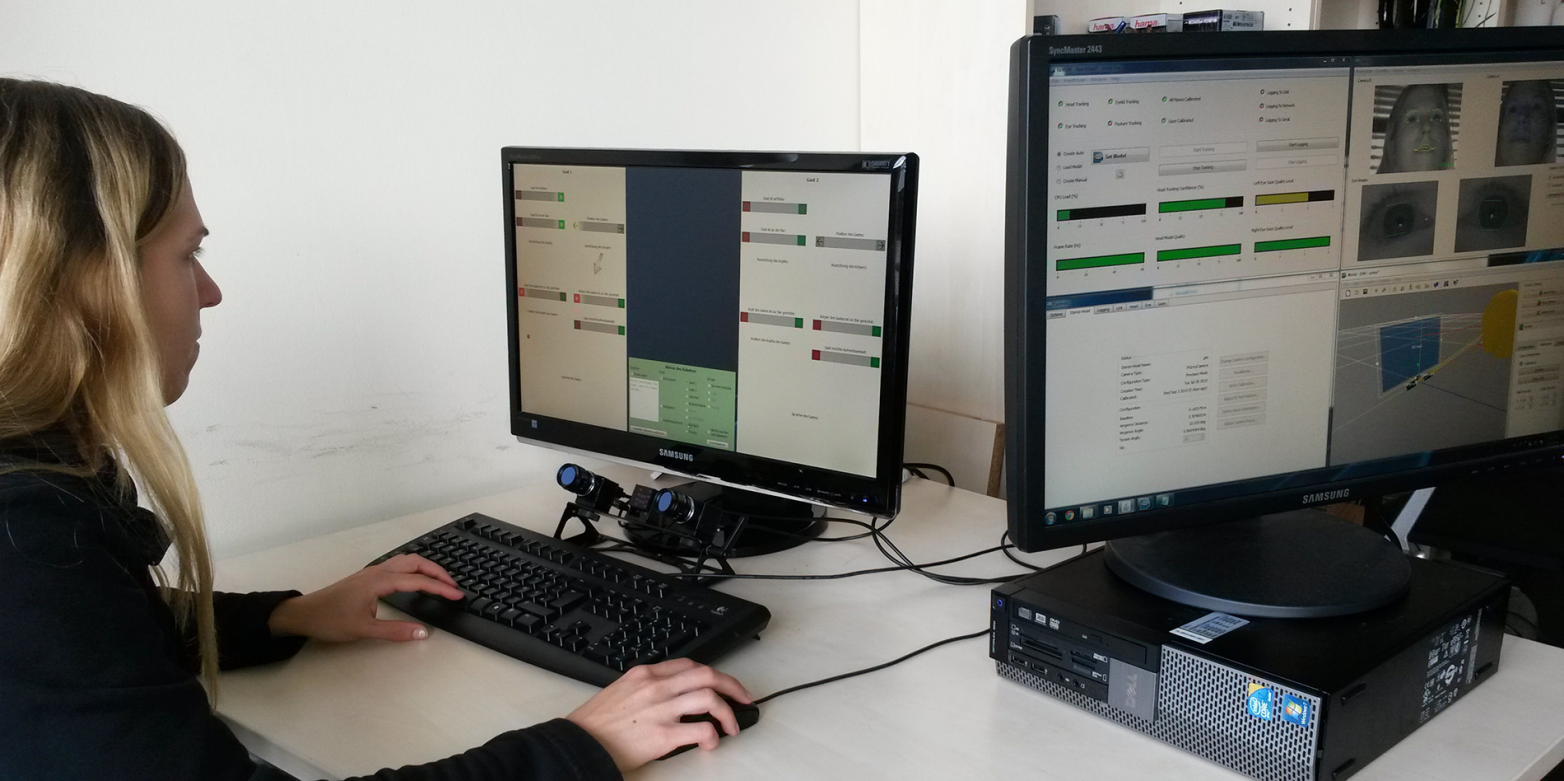
Setup of the Ghost-in-the-Machine (GiM) study with the ghost participant, eye tracker and GiM user interface on the left hand side, and the eye tracking control screen on the right hand side.

The participants were seated in front of a typical office computer screen (50 cm × 32 cm, 1920 × 1200 pixel) with a viewing distance of approximately 70 cm. Their eye gaze was recorded using a head-free faceLAB Eye Tracker (2009) positioned below the center of the screen. A dedicated JAVA application (Java Runtime Environment, 2012) presented the recognizer data and recorded the ghosts’ responses entered through a standard keyboard and mouse. We positioned the control screen for the eye tracker such that the participants could not see the display in order to avoid distraction. An experimenter checked whether the eye tracker worked as intended but was seated such that it was obvious to the participant that s/he was not observed. The setup is shown in **Figure 2.**

### Ghost-in-the-Machine Design

The ghosts were presented the output of the robotic recognizers by visualizing the variables using arrows and traffic lights. However, we were careful not to add any information that the planner cannot access. The ghosts responded to their customers by selecting actions from the robot’s repertoire that met their own expectations of appropriate behavior. In the following, we describe the control and information panels, their content and how this relates to a typical robotic architecture in more detail.

**FIGURE 3:**
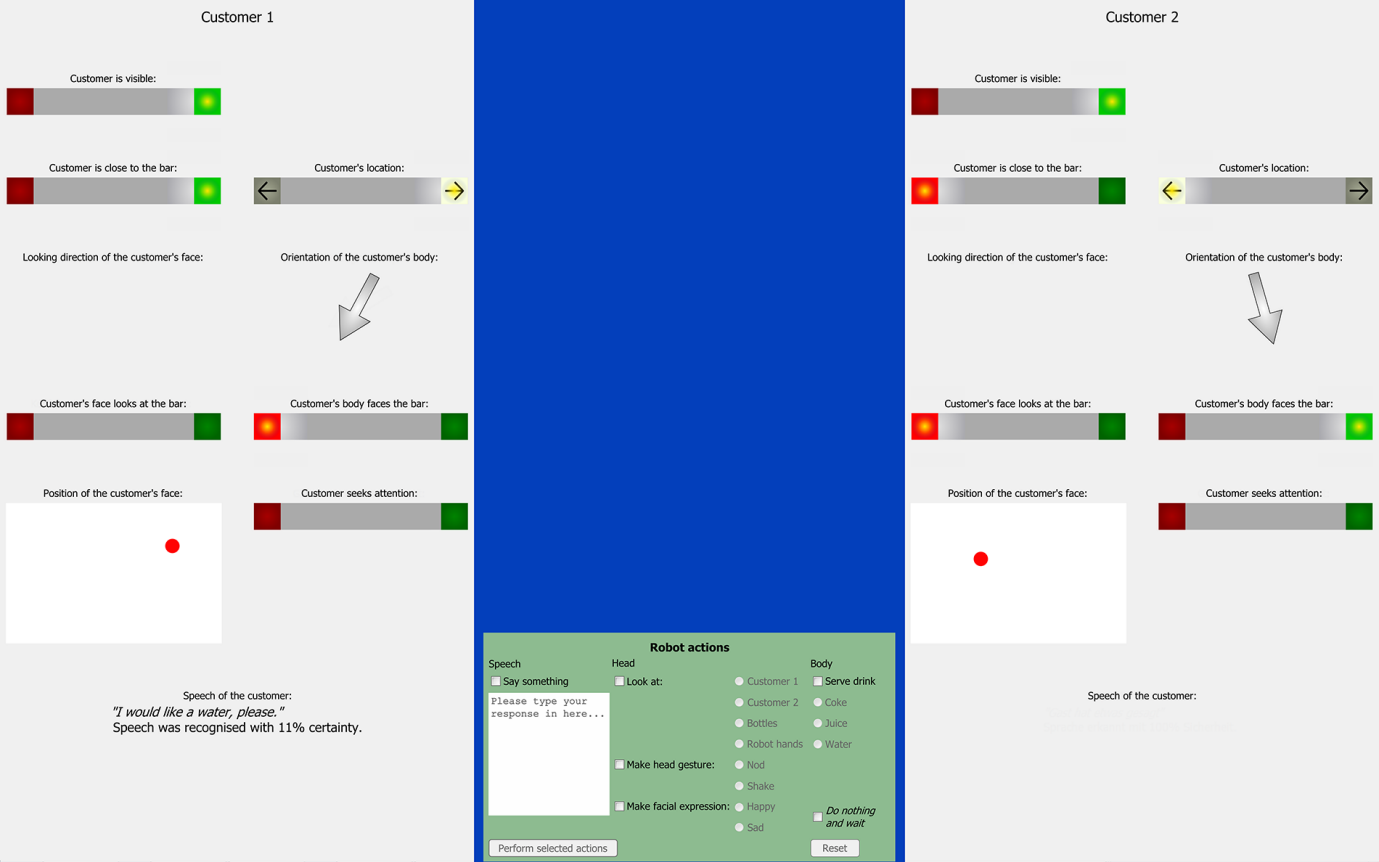
Full screen shot of the GiM interface showing information panels for up to two customers and the control panel for selecting actions.

The user interface for the ghosts consisted of three frames on a computer screen. On the left and right hand side of the screen, an information panel for each of the two customers was presented. At the bottom center of the screen, the control panel showed the robot’s repertoire (see **Figure 3**).

In the architecture of the robot, the sensors (e.g., camera or microphone) transmit their data to recognizers. These software programs analyze the raw data and extract information, e.g., the presence of a face or the words spoken. A component called the social state estimator collects these data and produces an updated state representation to the robotic planner if a major change was detected (Foster et al., 2013; Petrick and Foster, 2013). The updates slice the continuous data from the sensors and recognizers into distinct, temporally ordered updates of the scene. Each update formed a *turn* in this GiM study. A turn comprised of (a) an update of the information panels, and (b) a response by the ghosts. The next update was presented after the ghosts confirmed their selected actions (or explicitly selected no action) without time limit. Thus, the time span between recorded updates and presented updates could differ but their temporal order was unchanged. This turn-by-turn cycle continued until the trial was terminated. Since we used pre-recorded customer data in this study, the ghosts’ actions were recorded but never enacted by a robot and we did not try to convey otherwise. Thus, there was a potential discrepancy between the customer’s and ghost’s actions. This was addressed according to the experimental condition (see Materials and Conditions for details).

The user interface aimed at presenting the abstract recognizer data intuitively to the ghosts. The recognizer updates for each customer comprised of six binary variables (*is visible*, *is close to bar*, *location to left/right*, *face looks at bar*, *body faces bar*, *seeks attention*), three continuous numeric variables (*body orientation* and *face orientation* in degrees of angle, and the coordinates of the customer’s *face position*) and one variable dedicated to the customer’s speech. The values of the binary variables are computed by the social state estimator. For this, the social state estimator has built-in knowledge about the geometry of the robot’s bar and an interpretation mechanism that computes whether a customer is seeking the robot’s attention based on the his/her body posture and location at the bar (Foster et al., 2013).

The binary variables were presented in the style of a traffic light indicating that these variables could be true (green) or false (red). For the *customer’s location*, the same design was used with a left and right pointing arrow. If data for one indicator were not available, both lights were switched off. For example, if only one customer was visible in the scene, the other information panel was ‘switched off’. The angles of the body and face orientation (if available) were presented as arrows such that pointing downward represented a face/body looking straight at the counter. The position of the customer’s face was represented as a dot in a rectangle representing the space above the bar counter. Finally, the output of the ASR was presented at the bottom of each information panel. If speech was detected, this component showed the final speech hypothesis and its confidence level.

The control panel listed the robot’s repertoire in several groups with radio button selectors. The ghosts could use a free text field for speaking to the customers. The ghosts could make the robot look at one of the customers, the bottles, or the robot’s hands. They could select to nod or to shake the robot’s head and select a happy or a sad face. The panel also offered to serve one out of the three available drinks. Finally, the panel offered an option to do nothing and wait for the next update. This was a check box that had to be explicitly selected in order to hinder the ghosts from just clicking through the trials. In order to proceed to the next turn, the ghosts had to select at least one action or tick the *Do nothing and wait*-check box and confirm their selection. The interface hindered the ghosts from using impossible combinations (e.g., making a happy and a sad face at the same time). The selected action could be as complex as desired, e.g., looking at a customer, saying ‘Here is your drink,’ smiling and serving the drink.

### Materials and Conditions

The recognizer data were pre-recorded during an evaluation of the real robot in Munich (Foster et al., 2012). The evaluation trials included up to two customers in several configurations: both customers order drinks, one of the customers orders both drinks, and only one customer orders a drink with a bystander. For the evaluation, naïve participants were recruited and instructed to order a drink from the robotic bartender in English. The menu consisting of water, coke, and juice was introduced to them but no further instructions were given, i.e., there was no directive with regards on how to approach, speak to or take the drinks from the robot. After the participants placed an order, they evaluated the robot in a questionnaire (for further details, see Foster et al., 2012). Examples of the recognizer recordings are presented in the Supplementary Material.

This GiM study included an intention and a speech recognition condition. The intention recognition trials focussed on how interactions between customers and the robotic agent were initiated. We assessed the validity of the GiM paradigm by directly comparing its results to findings from an experiment with natural data (Loth et al., 2013). The speech recognition trials investigated how the ghosts identified which drink to serve. We were especially interested in socially appropriate repair strategies if the ASR hindered the robot from identifying an order or caused long delays.

We selected two practice and six experimental trials per condition. Based on the video recordings of the evaluation in Munich, the practice and two experimental trials per condition were selected to be relatively easy. That means that the recognizers provided clear data and in turn, the robot performed well without producing long delays or repeatedly asking the customers for their orders. The remaining four trials represented difficult cases that aimed at eliciting repair strategies. They included long delays in the interaction, failures to gather correct sensor data and/or failures of the robot’s decision policies. Alternating easy and difficult trials aimed at hindering the ghosts from treating less accurate data (e.g., very low confidence levels of the ASR) as if they were normal rather than thinking about strategies. All data presented to the ghosts were real recognizer data and thus, subject to noise, inaccuracies and sensor failures. This was explained to ghosts in the instructions in order to make clear that the displayed information was not ground truth but that data can be false or conflicting.

The intention and speech recognition trials differed in how the trials were organized. Since the main focus of the intention recognition trials was at the very beginning of the interaction, the respective recognizer data were presented starting from the first update of the recorded customer–robot interaction. Our aim was to establish how the ghosts identified whether a customer intended to place an order and how the existing computational account should be adapted. Since the indicator *Seeking attention* reflected the existing computation and could have biased the ghosts, it was switched off. The trials were terminated as soon as the ghost selected an action other than *Do nothing and wait*. Thus, we tracked when and how the ghosts first acknowledged a new customer. Since we used pre-recorded data, the ghosts may have selected an action that differed or occurred earlier than the robot’s actions during the evaluation. In turn, the customer’s response would not match the ghost’s actions. We minimized this risk by terminating the trials quickly. In contrast, the speech recognition trials aimed at a later stage in the interaction. Thus, we had to ensure that the ghost did not undertake any actions that mismatched with their customer until the order was placed. At the same time, the ghost had to be informed about what has happened until then. Thus, we presented the recognizer updates from the beginning but altered the control panel such that only a *Continue* button was available. Clicking *Continue* updated the indicators to the next update. As a result, the ghosts observed what has happened during the trial but were unable to deviate from the pre-recorded actions. As soon as one of the customers made a speech utterance, the control panel was rolled back and allowed the ghosts to select any of the actions. The trials terminated as soon as the ghost served a drink or there were no more updates to display. This possibly long interaction increased the risk of a discrepancy between the ghost and its customers. However, we suspected that the ghosts would aim to understand the drink order and we knew from the recordings that the customers repeated their drink order in various ways. We report on this in the results and discussion sections. In all trials, the ghost was informed about the end of a trial by a pop-up message and removing all panels from screen. Once the message was confirmed, the panels appeared on screen and the next trial started.

## Results

We report the results of the intention and speech recognition trials separately. In each section, we summarize (a) the recognizer data displayed in the information panels, (b) the turn duration and summed dwell time on screen, (c) the dwell time on each indicator, and (d) the selected response. The analyses of the recognizer and eye tracking data refer to the addressed customer. For example, if the ghost selected to look at *Customer 1*, the recognizer data of *Customer 1* were analyzed.

In general, the ghosts experienced the experiment as very immersive. This was the case even though the customer data were pre-recorded, there was no actual customer feedback and the interface was comparably simple. For example, some ghosts apologized for having made jokes to their customers after the experiment or complained about the unresponsiveness of their customers if the recognizers did not show any new information. However, some ghost participants trialed how well our design would respond to unexpected behaviors and tried to take advantage of the pre-recorded nature of the data. These trials are listed in detail with regards to each condition.

### Intention Recognition Trials

The experiment comprised a total of 174 intention recognition trials from 29 participants. Three ghosts repeatedly selected *Do nothing and wait* until the ASR unequivocally identified an order. Their data and two additional trials showing a similar pattern were excluded (in total 20 trials, 11.5%). One additional trial was excluded because the ghost ignored the customers and did not respond at all. Thus, the following analyses cover the 153 remaining trials. Each trial was a customer–staff interaction of one or more turns. The customers’ actions were presented through an update of the indicators and the ghosts responded by selecting a robot action which completed the turn. The intention recognition trials continued with another turn if the ghosts selected *Wait and do nothing* (*No response*) and were terminated with the ghosts’ first selected action (*Response*). Thus, each of the 153 trials comprised one *Response* turn. The total of 117 *No response*-turns distributed over 69 trials.

#### Recognizer Data

The data in **Table 1** summarize the recognizer data by listing the state of the traffic light and arrow indicators as well as the presence of detected speech in the information panel of the addressed customer (see **Figure 3**). Please note that the indicator *Seeks attention* was switched off in all intention recognition trials (see Materials and Conditions). The arrow shaped indicator showing the *Face orientation* was never active due to a technical problem during the data acquisition. For the same reason, the binary indicator *Face to bar* either showed no value or *false*, i.e., this indicator never showed *true*. Thus, the information from both indicators was potentially misleading and we return this when discussing the results.

**Table 1:** State of the indicators of the addressed customer as a function of whether the ghosts acknowledged the new customer (Response) or not (No response).

Indicator	State	No response		Response	
			
		Number	Percent	Number	Percent
Visible	Unknown	48	41%	4	3%
	False	2	2%	2	2%
	True	67	57%	147	95%
Close to bar	Unknown	48	41%	4	3%


	False	42	36%	52	34%
	True	27	23%	97	63%
Location	Unknown	48	41%	4	3%
	Known	69	59%	149	97%
Body orientation	Unknown	58	50%	19	12%


	Known	59	50%	134	88%
Face orientation	Unknown	117	100%	153	100%


	Known	0	0%	0	0%
Body to bar	Unknown	48	41%	4	3%


	False	56	48%	57	37%
	True	13	11%	92	60%
Face to bar	Unknown	48	41%	4	3%


	False	69	59%	149	97%
	True	0	0%	0	0%
Seeks attention	Unknown	117	100%	153	100%


	Known	0	0%	0	0%
Face position	Unknown	51	44%	11	7%


	Known	66	56%	142	93%
Speech	Said nothing	117	100%	153	100%
	Said something	0	0%	0	0%


The continuous indicator *Body orientation* was recoded as a binomial variable such that we distinguished whether the arrow was displayed (*known*) or not (*unknown*). We opted for this simplification because the recognizer was only able to compute the angles from the camera image if the customers faced the camera to some degree. If the customer turned away especially when turning outward, the recognizer could not determine the angle. The recorded angles ranged between 76° and –36°, i.e., the indicator never showed that a customer was turned away from the bar. Thus, by recoding the variable into *known* and *unknown*, we created a very lenient version of the *Body to bar* indicator. Entering both variables in the analysis allowed us to distinguish whether a stricter metric as applied by the social state estimator for the *Body to bar* variable or a more lenient coding as in the recoded *Body orientation* variable was more appropriate. Similarly, we recoded the *Face position* indicator’s values into *known* and *unknown*. This indicator was only active if the customer’s face was directed toward the bar and if it was within the observable area in front of the camera.

By grouping the state of the indicators into *No response* and *Response* updates, the data in **Table 1** shows a summarized history of the trials. The ghosts acknowledged customers in the *Response* turns whom they have not acknowledged in the preceding *No Response* turns. Thus by identifying how these groups differ, we can understand which indicator changes were crucial to the ghosts to initiate a customer–staff interaction. Most of the indicators were highly interdependent, e.g., the body orientation could only be measured if the customer was visible to the system. Thus, we designed a multinomial regression model using the *nnet* package (Ripley and Venables, 2014) of R development core team (2007). The binary variable distinguishing between *Response* and *No response* was used as the dependent variable and the variables coding the state of the indicators (see **Table 1**) served as predictors (independent variables). Thus, the regression used the state of all indicators to distinguish whether an update was part of the history (*No response*) or whether it triggered an acknowledgment (*Response*). The predictor variables were excluded from the regression model if the more parsimonious model did not differ statistically significantly from the full model. Thus, only the set of predictors that could distinguish most effectively between a *No response* and a *Response* turn would remain in the model, i.e., the indicators that had the greatest influence in the ghosts’ decision.

The data in **Table 1** show that the customers never said anything, i.e., the ghosts always acknowledged the customer before s/he said something. Thus, the speech was excluded as triggering the ghosts’ response and did not enter the regression model. After excluding all predictors but the *Close to bar*, *Body to bar*, and the *Face position* indicators, the multinomial model had a *Cox and Snell R*^2^= 0.334 compared to *R*^2^= 0.335 of the full model. Excluding the *Face position* resulted in a statistically significant difference. But the model based on the *Close to bar* and the *Body to bar* indicators explained almost as much of the variance *R*^2^= 0.321 as the model including these three variables^2^. We concluded that the *Close to bar* and the *Body to bar* indicators had the greatest impact on the ghosts’ decisions in the intention recognition trials.

#### Turn Duration and Dwell Time

The user interface measured the time span between an update and the corresponding response of the ghost, i.e., the time required to complete a turn (see **Table 2**). The data reflect a comparison of 153 acknowledgments (*Response*) and 117 intermediate updates (*No response*). If a trial included several intermediate turns, their duration and dwell times were averaged before entering the analysis. Thus, 69 intermediate updates contributed to the turn duration. Three trials (one *No response* and three *Response* turns) were excluded from the analysis of the dwell times because the eye tracker was unable to record any data. The dwell times were determined by mapping the point of gaze and duration provided by the faceLAB software to the components of the display. The dwell time on the control panel is possibly underestimated due to its position at the bottom center of the screen. First, the noise of the eye tracker could have resulted in falsely detecting gazes at lower parts of the panel as outside the screen. Secondly, glasses are more likely to reflect the IR illuminator such that the eyes are covered by the reflection if the participant looks straight toward the illuminator below the center bottom region of the screen. However, this design allowed us to position the information panels that we analyzed in more detail with a maximum distance to this area.

**Table 2:** Average turn duration, dwell time on the information and control panels as a function of whether the ghost acknowledged a new customer (Response) or not (No response).

Time	No response		Response	
		
	*Time in ms*	*SD in ms*	*Time in ms*	*SD in ms*
Turn duration	12728	6540	18105	10095
Dwell time addressed customer	2179	1988	3774	3017
Dwell time other customer	2304	1870	828	1145
Dwell time on control panel	2089	1741	4609	3956


The turn duration and dwell times were analyzed with JASP (Love et al., 2014). We report the *BayesFactors* from a Bayesian *t*-test (Rouder et al., 2009; Morey et al., 2014) alongside the respective standard *t*-test statistics. A Cauchy distribution with scale parameter $\frac{1}{\sqrt{2}}$ served as the prior for the effect size (see Rouder et al., 2009). The advantage of using Bayesian *t*-tests is that they also allow researchers to evaluate the amount of evidence for the null hypothesis, which is not possible with standard, frequentist statistical tests. The effect sizes of the standard *t*-tests were computed using G^∗^Power (Faul et al., 2007). The independent samples comparison of the turn durations showed that if the ghosts acknowledged a customer they took statistically significantly longer compared to selecting to wait for the next update [*t*(220) = 4.054, *p* < 0.001, *BF*_10_ = 246.6, *d* = 0.57]. Also, the ghosts dwelled longer on the information panel of the customer whom they addressed [*t*(216) = 3.983, *p* < 0.001, *BF_10_* = 190.2, *d* = 0.56] and the control panel [*t*(216) = 5.033, *p* < 0.001, *BF*_10_ = 12765, *d* = 0.70] if they acknowledged the new customer. In contrast, the ghosts attended the information of the other customer less if they made an acknowledgment [*t*(216) = 7.158, *p* < 0.001, *BF*_10_ = 6.03^∗^10^8^, *d* = 0.94].

#### Dwell Time on Indicators

The data in **Table 3** summarize the ghosts’ dwell times on each indicator of the information panel corresponding to the addressed customer. For accommodating the absolute differences in turn duration, we computed the relative dwell time on each indicator by normalizing with the summed dwell time of the respective information panel (see **Table 2**).

**Table 3:** Mean dwell times for each indicator of the addressed customer as a function of whether the ghosts acknowledged a new customer (Response) or not (No response).

Indicator	No response				Response			
		
	*Dwell time in ms*	*SD in ms*	*Relative dwell time*	*SD in pp*	*Dwell in ms*	*SD in ms*	*Relative dwell time*	*SD in pp*
Visible	328	513	15.5%	17.9	399	678	11.2%	17.3
Close to bar	367	545	16.4%	16.9	485	775	11.2%	12.7
Location	181	268	7.3%	9.2	329	543	10.8%	15.0
Body orientation	160	246	6.1%	8.3	290	407	9.8%	14.4
Face orientation	68	90	4.0%	5.8	100	174	2.4%	4.2
Body to bar	401	491	18.2%	14.5	816	983	20.7%	18.5
Face to bar	205	301	9.8%	10.2	395	609	9.1%	12.1
Seeks attention	191	384	9.2%	12.1	386	560	10.7%	12.9
Face position	239	343	12.6%	15.2	520	887	12.3%	17.2
Speech	16	45	0.9%	2.1	56	109	2.0%	5.8


We analyzed which indicators received more or less of the ghosts’ attention in the *Response*-turns, i.e., the relative dwell times during their decision to acknowledge a new customer. If the ghosts looked randomly at the information panel, we would expect an even distribution of the relative dwell time of 0.1 across the ten indicators. Thus, one-sample *t*-tests and corresponding Bayesian tests were performed against an expected mean of 0.1. There was a statistically significant difference for the *Body to bar*-indicator [*t*(149) = 7.061, *p* < 0.001, *BF*_10_ = 1.23^∗^10^8^, *d* = 0.58] indicating that the ghosts attended this indicator longer than expected. The *Face orientation* [*t*(149) = 22.466, *p* < 0.001, *BF*_10_= 2.20^∗^10^46^, *d* = 1.81] and the *Speech* indicators [*t*(149) = 17.076, *p* < 0.001, *BF*_10_= 4.58^∗^10^33^, *d* = 1.39] were avoided compared to a random gaze pattern. There was no statistical difference for all other indicators [all *t*(149) < 2.0, all *p* > 0.05] and the *BayesFactor* indicated their relative dwell times were equal to a random distribution (all *BF*_10_ < 0.3).

#### Responses

The ghosts acknowledged their customers by selecting a response from the control panel (see **Figure 3**). The options that the ghosts selected in 153 trials are summarized in **Table 4.**

**Table 4:** Number and percent of the selected actions for acknowledging a new customer.

Action	Number	Percent
Say something	40	26%
Greeting	25	63%
Prompt to order	19	48%
Looking at something	142	93%
At customer	136	96%
At bottles	4	3%
At hands	2	1%
Make head gesture	4	3%
Nodding	4	100%
Shaking	0	0%
Make facial expression	59	39%
Happy	58	98%
Sad	1	2%
Serve a drink	0	0%


In the vast majority of cases, the ghosts selected to look at their customer and in one third of the cases made a happy face. Only one quarter of the responses included a verbal utterance. This was either a greeting (e.g., “Hello?”), a prompt to place an order (e.g., “What would you like?”), or both.

### Speech Recognition Trials

In total 174 speech recognition trials were presented to 29 participants. In two trials, the ghost did not serve a drink and the trial was terminated after the pre-recorded customer data ran out. These trials were excluded from all further analyses. In sum 172 drinks were served (one per valid trial) and 553 intermediate updates and their corresponding *No serving*-responses (turns) were recorded. They were distributed unevenly across the trials: *M* = 3.2, *SD* = 5.9, *Mdn* = 1.0, *Max* = 38, *Min* = 0. In 12 trials the ghosts served a drink in their first response. Thus, *No serving*-responses occurred in 160 trials. In 98 trials one *No serving*-response occurred and another 37 trials included three *No serving*-responses.

#### Recognizer Data

The recognizer data of the addressed customer in the speech recognition trials are summarized in **Table 5.** These recognizer updates were either followed by the ghost serving a drink (*Serving*) or the ghost decided to continue the interaction without a serving (*No serving*), e.g., by asking a question. Please note that the *Face orientation* and *Face to bar indicators* did not work as a result of a failure to record the data during the evaluation. The variable *Body orientation* was recoded into *known* and *unknown* as in the intention recognition trials. The range of the recorded angles was between 22° and –59° and was smaller compared to the intention recognition trials.

**Table 5:** State of the indicators of the addressed customer as a function of whether the ghosts served a drink.

Indicator	State	No serving		Serving	
			
		Number	Percent	Number	Percent
Visible	Unknown	1	0%	1	1%
	False	95	17%	54	31%
	True	457	83%	117	68%
Close to bar	Unknown	1	0%	1	1%
	False	80	14%	32	19%
	True	472	85%	139	81%
Location	Unknown	1	0%	1	1%
	Known	552	100%	171	99%
Body orientation	Unknown	8	1%	5	3%
	Known	545	99%	167	97%
Face orientation	Unknown	553	100%	172	100%
	Known	0	0%	0	0%
Body to bar	Unknown	1	0%	1	1%
	False	250	45%	85	49%
	True	302	55%	86	50%
Face to bar	Unknown	1	0%	1	1%
	False	552	100%	171	99%
	True	0	0%	0	0%
Seeks attention	Unknown	1	0%	1	1%
	False	14	3%	5	3%
	True	538	97%	166	97%
Face position	Unknown	1	0%	1	1%
	Known	552	100%	171	99%
Speech	Said nothing	375	68%	18	10%
	Greeting	100	18%	0	0%
	Order	78	14%	154	90%


The data in **Table 5** compare the state of all indicators when the ghosts served a drink to an average of earlier updates during the course of their interaction. This comparison can identify which change in the available information made the ghosts serve a drink. The data show that the customers were almost always detected as seeking attention, their face position was known and a speech utterance was recognized when the ghosts served a drink. The majority of customers was close to the bar. But the data also suggest that customers were less likely to be served if they were visible. In order to determine which of the indicators influenced the ghosts’ decision to serve a drink (*No serving* vs. *Serving*), we designed a multinomial regression model using the state of all indicators as predictors and eliminated them if the more parsimonious model did not differ significantly from the full model. This regression model aimed at identifying the indicators that can distinguish most effectively between an update that occurred at some point in the interaction and the update that triggered the ghosts to serve a drink. After removing all predictors but the *Body orientation* and the *Speech* indicators, the multinomial model had a *Cox and Snell R*^2^= 0.266 compared to *R*^2^= 0.269 of the full model. Removing the *Body orientation* indicator resulted in a statistically significant difference, but the loss of explained variance was about one percent *R*^2^= 0.256. We concluded that the customer’s speech had the greatest impact on the ghost’s decision to serve a drink.

The customer’s speech was presented together with the confidence level of the ASR. We compared the confidence levels of the customers’ orders (*N*_total_ = 232, *M*_total_ = 49.43, *SD*_total_ = 30.03, *Mdn*_total_ = 42.00) when the ghosts served a drink (*N*_serving_ = 154, *M*_serving_ = 59.73, *SD*_serving_ = 28.54, *Mdn*_serving_ = 73.00) and when they did not (*N*_noserving_ = 78, *M*_noserving_ = 29.09, *SD*_noserving_ = 21.35, *Mdn*_noserving_ = 24.00). This reflects a comparison of the 78 orders without a serving and the 154 servings in the bottom row of **Table 5.** The independent samples test revealed a statistically significant difference [*t*(230) = 8.366, *p* < 0.001, *BF*_10_= 1.01^∗^10^12^, *d* = 1.02] indicating that the confidence level was higher when the ghosts served a drink compared to when they did not.

#### Turn Duration and Dwell Time

The turn durations (time between update presented on screen and response) are presented in **Table 6.** The data reflect a comparison of 172 servings (*Serving*) and 553 intermediate updates (*No Serving*). If a trial included several intermediate turns, the duration and dwell times were averaged before entering the analysis such that 160 data points contributed to *No serving*-data.

**Table 6:** Average turn duration, dwell time on the information and control panels as a function of whether the ghost served a drink.

Time	No serving		Serving	
		
	*Time in ms*	*SD in ms*	*Time in ms*	*SD in ms*
Turn duration	25374	15632	25250	16809
Dwell time addressed customer	3083	1561	2623	2664
Dwell time other customer	1540	2066	791	1094
Dwell time on control panel	7081	6149	8754	7496


The turn duration and dwell times were analyzed as above. The independent samples comparison of the turn durations showed that there was no statistically significant difference between servings and intermediate updates [*t*(330) = 0.069, *p* = 0.945, *BF*_10_= 0.087]. Also, there was no such difference in the dwell time on the information panel of the addressed customer [*t*(330) = 1.599, *p* = 0.111, *BF*_10_= 0.302]. However, the ghosts dwelled statistically significantly less on the information panel of the other customer if they served a drink [*t*(330) = 4.167, *p* < 0.001, *BF*_10_= 350.9, *d* = 0.45]. There was a tendency indicating that the ghosts attended the control panel longer if they served a drink [*t*(330) = 2.214, *p* = 0.028, *BF*_10_= 0.941, *d* = 0.24]. The *t*-test indicated a statistically significant difference. But the *BayesFactor* did not and the effect size was comparably small. Thus, we do not consider this difference as significant.

#### Dwell Time on Indicators

The eye tracking data were analyzed as in the intention recognition trials. The data in **Table 7** reflect the information panel of the addressed customer. The data and analyses below refer to the average of 172 servings and intermediate updates in 160 trials.

**Table 7:** Mean dwell times for each indicator of the addressed customer as a function of whether the ghost served a drink.

Indicator	No serving				Serving			
		
	*Dwell time in ms*	*SD in ms*	*Relative dwell time*	*SD in pp*	*Dwell in ms*	*SD in ms*	*Relative dwell time*	*SD in pp*
Visible	161	354	4.5%	7.7	131	344	2.7%	5.1
Close to bar	161	375	4.4%	6.7	198	465	5.3%	11.0
Location	189	268	9.2%	15.5	166	315	7.9%	16.7
Body orientation	167	254	5.2%	6.1	111	233	5.4%	15.3
Face orientation	57	120	2.1%	5.4	53	157	2.1%	8.8
Body to bar	481	591	16.7%	17.5	331	581	10.8%	15.0
Face to bar	130	215	4.2%	8.4	116	299	3.5%	9.5
Seeks attention	428	517	16.1%	17.2	316	316	11.7%	16.0
Face position	608	974	17.3%	20.5	560	980	21.3%	29.9
Speech	700	1093	20.3%	26.6	641	807	29.4%	33.9


The relative dwell times of the *Serving*-turns were analyzed as above using a one-sample *t*-test against a mean value of 0.1 across the ten indicators. The ghosts attended the indicators *Visibility* [*t*(171) = 18.791, *p* < 0.001, *BF*_10_= 1.36^∗^10^40^, *d* = 1.43], *Close to bar* [*t*(171) = 5.642, *p* < 0.001, *BF*_10_= 1.22^∗^10^5^, *d* = 0.43], *Body orientation* [*t*(171) = 3.939, *p* < 0.001, *BF*_10_= 97.379, *d* = 0.30], *Face orientation* [*t*(171) = 11.832, *p* < 0.001, *BF*_10_= 7.34^∗^10^20^, *d* = 0.90], and *Face to bar* [*t*(171) = 9.081, *p* < 0.001, *BF*_10_= 2.06*10^13^, *d* = 0.68] statistically significantly less than expected by a random distribution. The relative dwelling times on the indicators for *Location* [*t*(171) = 1.646, *p* = 0.102, *BF*_10_= 0.230], *Body to bar* [*t*(171) = 0.683, *p* = 0.495, *BF*_10_= 0.076] and *Seeking attention* [*t*(171) = 1.374, *p* = 0.171, *BF*_10_= 0.154] did not differ from a random distribution. In contrast, the indicators for the *Face position* [*t*(171) = 4.982, *p* < 0.001, *BF*_10_= 6113.811, *d* = 0.38] and the *Speech* [*t*(171) = 7.497, *p* < 0.001, *BF*_10_= 1.88^∗^10^9^, *d* = 0.57] received more attendance than at random. It should be noted that the face coordinates of *Customer 2* were closely located to the control panel. Their distance was the shortest on the entire screen. Thus, if the ghosts dwelled on the serving options of the control panel a misattribution of the point of gaze could occur between the panel and the *Face position* of *Customer 2* but not of *Customer 1*. The difference in the relative dwell times of this indicator of *Customer 1* [*M* = 16.7%, *SD* = 26.8pp] and *Customer 2* [*M* = 36.9%, *SD* = 34.2pp] during the *Serving*-turn supported this assumption. Thus, we repeated the one-sample analysis in Servings to *Customer 1* only [*t*(131) = 2.844, *p* = 0.005, *BF*_10_= 3.392, *d* = 0.25]. After excluding the potentially misattributed points of gaze, the effect size was smaller but the result was still compatible with our initial analysis indicating that the ghosts dwelled longer on the *Face position* indicator than expected with a random distribution.

The greater number of intermediate turns in the speech recognition trials allowed us to address whether the ghosts’ attention changed in terms of relative dwell times during the course of the trials. We compared the relative dwell times of the information panel of the addressed customer in the *No serving* and *Serving*-turns. An independent samples test showed that the relative dwell time on the *Speech* was larger in the *Serving*-turns [*t*(330) = 2.714, *p* = 0.007, *BF*_10_= 3.082, *d* = 0.29]. The relative dwell times reduced for the *Body to bar* indicator [*t*(330) = 3.329, *p* < 0.001, *BF*_10_= 18.173, *d* = 0.36]. This tendency was also found in the *Visibility* [*t*(330) = 2.482, *p* = 0.014, *BF*_10_= 1.726, *d* = 0.26] and *Seeks attention* indicators [*t*(330) = 2.426, *p* = 0.016, *BF*_10_= 1.512, *d* = 0.28]. In these cases, the *t*-test showed a statistically significant difference but the *BayesFactor* was not conclusive. There was no statistical difference for all other indicators [all *t*(330) < 2.0, all *p* > 0.05] and the *BayesFactor* provided evidence in favor of the relative dwell times being equal in *Serving* and *No serving*-turns (all *BF*_10_ < 0.3).

#### Responses

The ghosts responded to their customers by selecting a response from the control panel (see **Figure 3**). The data in **Table 8** summarize the responses to 553 intermediate updates (*No serving*) and 172 servings.

**Table 8:** Number and percent of the selected actions during the interaction and accompanying the serving of a drink.

Response	No serving		Serving	
		
	*Number*	*Percent*	*Number*	*Percent*
Say something	290	52%	121	70%
Greeting	48	17%	0	0%
Prompt to order	118	41%	0	0%
Prompt to repeat	123	42%	0	0%
Confirming serving	17	6%	116	96%
Looking at something	395	71%	161	94%
At customer	390	99%	156	97%
At bottles	4	1%	4	2%
At hands	1	0%	1	1%
Make head gesture	59	11%	77	45%
Nodding	49	83%	77	100%
Shaking	10	17%	0	0%
Make facial expression	209	38%	121	70%
Happy	199	95%	119	98%
Sad	10	5%	2	2%
**Total**	553		172	


The ghosts made the robot look at the customer in the majority of their responses. In particular, when serving a drink almost all ghosts selected that the robot should look at the customer. About half the responses were accompanied by speech during the interaction. These utterances were mainly prompting the customers either to place an order (e.g., “What would you like?”) or asking the customers to repeat their order using one out of two strategies. First, the ghosts just asked their customer to repeat their utterance (e.g., “Could you say this again?”). Secondly, they repeated the name of the drink that the ASR presented as the most likely guess (e.g., “A water for you?”). Both strategies were used in about half of the cases (see **Table 9**). The ghosts used similar utterances when serving a drink. Either they said something friendly to confirm that the order is about to be served (e.g., “Here you are.”) or they included the name of the drink in their utterance (e.g., “Here is your water.”). Both options were used in about half the cases (see **Table 9**). The servings were also accompanied by more expressive face and head movements. Two thirds of the servings included a happy face compared to only one third of the intermediate responses. Also, almost half the servings included a nodding but only 10% of the intermediate responses was accompanied by a head gesture at all.

**Table 9:** Number and ratio of echo questions and statements in prompts to repeat the order and confirmations to serve the drink as a function of whether the ghosts served a drink.

Response	No serving		Serving	
		
	*Number*	*Percent*	*Number*	*Percent*
Prompt to repeat	123		0	
* Echo question*	*56*	*46%*	*0*	*0%*
Confirming serving	17		116	
* Echo statement*	*2*	*12%*	*52*	*45%*


The data in **Table 8** suggest that in 17 trials the serving of a drink was verbally confirmed but not actually served. Thus, we inspected these cases more closely. In eight cases, the drink was served in the next turn, i.e., not immediately after confirming the order but at the next opportunity. The other nine trials involved an utterance that is ambiguous if used without punctuation marks (“Bitteschön”). In **Table 8**, this was categorized as a polite German confirmation (“Bitteschön.” [Here you are.]). This would imply that the ghosts have forgotten to serve the drink. However, in the seven cases that did not repeat the name of the drink, it could also invite to place an order (“Bitteschön?” [What can I do for you?]). This implies that the ghosts ignored the customer’s utterance and used an expression for inviting to place an order out of the blue. We cannot decide whether one of these interpretations was intended by the ghosts. But the closer analysis showed that the ghosts rarely used a verbal confirmation to serve a drink without actually serving it. In all cases, the trial continued until the ghost served a drink. It should be noted that the customers had to repeat their orders several times with the real robot. Thus, if the ghosts did not use the next turn for serving a drink, they served it with another drink order later in the trial.

## Discussion

Most of the ghosts reported that they experienced GiM as very immersive and experienced a turn-by-turn role-game. They invested great efforts into establishing a social interaction with their customers despite the fact that their behavior was pre-recorded and displayed in a number of indicators. First, the number of trials and the time spent illustrates the ghosts’ efforts. The majority of trials involved three or less turns but the ghosts used up to 38 turns if necessary. The variance in the number of turns illustrates that they adapted to each customer in order to entertain a socially credible interaction. Secondly, the human ghosts were more efficient on the same data than the actual robot. Since each trial of the pre-recorded data had a maximum number of turns defined by the original robot–customer interaction, the ghosts would have been unable to complete the trial if they required more data than the robot. This occurred only three times compared to the 325 trials that entered our analyses. During the evaluation, the robot had a real-time interaction with its customers such that it could ask questions and elicit a direct response. In contrast, the ghosts communicated with pre-recorded customers whom could not respond to, e.g., a clarification question. Thus, the ghosts used their social knowledge to outperform the robotic bartender, e.g., the ghosts’ responses indicated that they interpreted their customers’ responses in the context of their own questions and utterances which were not present at the time of recording. Thirdly, the results of the intention recognition trials are compatible with findings from observations in the real world and experiments using natural stimuli. Thus, we conclude that the ghosts made credible efforts and that the results reflect human social behavior that can reveal strategies for improving human–robot interactions. We discuss the results in more detail starting with the intention recognition trials and secondly, the speech recognition trials.

### Intention Recognition Trials

The results of the intention recognition trials showed that ghosts relied on the *Close to bar* and *Body to bar* indicators for identifying new customers. This finding replicates the results of an experiment using natural videos and snapshots from real bars where the participants detected that customers bid for the attention of bar staff if they were close to the bar and their body and/or head was directed to the bar (Loth et al., 2013). This behavior was also observed at ticket counters in Amsterdam Centraal station. Similar to the bartending robot, a member of staff sits at a fixed position behind the counter and waits for customers. The interactions were initiated if a customer approached the counter and looked at the assistant (Brouwer et al., 1979; Clark, 2012). As in our results, the distance to the counter and head/body direction were essential in this setting. That means that the interactions were initiated by the placement (Clark, 2003) of the customer’s body. More specifically, this was described as asking a wordless question (Clark, 2012). Furthermore, implementing this strategy for detecting customers with the intention to place an order produced more reliable and more stable results than other classifiers (Foster, 2014). Thus, the social signal for initiating an interaction is formed by these two components. The results of this GiM study supported that finding and demonstrated that we can obtain reliable and valid results with this paradigm (also see Loth et al., 2014).

The ghosts’ detection strategy relied on only two recognizer modalities (distance to bar and body orientation) whereas other modalities were not relevant including the customer’s speech. However, this finding could be attributed to the customer’s speech being (a) irrelevant, or (b) relevant, but there was no speech detected during the data recording and speaking coincided with other cues in the natural data experiments. The design of this GiM study enabled us to distinguish between these possibilities. First, in the natural data experiment the participants had to judge whether a particular snapshot showed a customer bidding for attention. In contrast, the control panel of the GiM interface offered the ghosts to wait for another update that may include additional cues such as a speech utterance. Thus, the ghosts decided when they responded to a new customer. However, the ghosts never waited for a speech utterance. Secondly, the eye tracking data allowed us to identify which recognizer modality was attended by the ghosts. They dwelled on the *Speech* indicator less than expected with a random gaze pattern. Rather, they focussed on the information about the customer’s pose and position, especially the binary indicator *Body to bar*. It could be argued that the ghosts did not gaze at the *Speech* indicator because speech was not displayed and thus, they looked at something else. But this was not the case. The *Seeks attention* indicator would have provided a straight forward hint for the ghosts but it was disabled. Hence, the *Seeks attention* and *Speech* indicators equally showed no information. However, the ghosts dwelled on the potentially relevant *Seeks attention* indicator about 10% of their relative dwell time but only 2% on the *Speech* indicator (see **Table 3**). Thus, the ghosts deliberately ignored the *Speech* indicator whereas there was no clear pattern of ignoring or focussing on the *Seeks attention* indicator. Together, this provided converging evidence that modalities other than the distance to the bar and the head/body orientation were not relevant for detecting the intention to place an order, and generally to initiate an interaction.

Using this strategy indicates that the ghosts subconsciously accessed their knowledge of initiating an interaction and specifically scanned the panels for the expected social signal. In turn, they could ignore most of the recognizer data without risking to ignore a customer. However, it appears counter-intuitive to ignore information since there was no time pressure that could have hindered the ghosts from scanning the entire display. This could be attributed to the fact that the human cognitive resources are limited in general (Broadbent, 1969) and in particular within one sensory modality such as vision (Allport et al., 1972; Mcleod, 1977). Thus, the ghosts used their social knowledge for limiting the information that they attended to a few relevant indicators. For example, the GiM interface included two indicators for the customer’s body orientation. The arrow shaped indicator *Body orientation* provided an analog display of recognizer data and was larger than the *Body to bar* indicator which depicted a binary value computed by the social state estimator. Despite the fact that the binary indicator was smaller on the display, the ghosts attended and relied on this to a greater extent compared to the analog version. First, one of the indicators was sufficient and thus, the ghosts limited their attention to one of them. Secondly, the ghosts consistently selected the binary indicator. One of the differences between the two indicators is the required effort for using the information. For interpreting the arrow indicator the ghosts would have to evaluate the angle of the customer’s body orientation themselves whereas the binary indicator was simpler and provided this interpretation.

The ghosts not only ignored redundant information and relied on the most convenient display, they also ignored irrelevant data. The results showed that they almost exclusively focussed on the customer’s distance to the bar and their body orientation. This pattern was not an artifact of our GiM design. For example, the participants in the natural data experiment only analyzed the body posture of customer who were close to the bar but not of other customers (Loth et al., 2013). A similar focus on task-relevant aspects was observed in intentional blindness in the visual (Simons and Chabris, 1999) and auditory domain (Dalton and Fraenkel, 2012). Thus, focussing on those aspects that are relevant for detecting an expected social signal reflects general cognitive processes in social interactions. Identifying these strategies is crucial for human-robot interaction as it allows to discard possibly misleading data, e.g., a speech utterance from another customer. Using these social strategies saves computational effort, improves the robot’s reliability and makes its performance more predictable by being more human-like.

The GiM paradigm also allows the manipulation of very specific pieces of information, e.g., for investigating the relevance of a particular modality and for eliciting recovery strategies in sensor failures. The customer’s face data were not recorded during the robot evaluation resulting in an apparent sensor failure. Thus, the indicator *Face orientation* never worked and the binary *Face to bar* indicator either indicated that the face was not detected or that it did not look toward the bar. Thus, attending and using this information could have misled the ghosts. They could have assumed that the customer looked away from the bar and has not intended to interact with them. However, the ghosts did acknowledge their new customers. Thus, we concluded that the ghosts recognized that the face related information was unreliable, discarded this information and recovered from that sensor failure by relying on data about the customer’s body instead, specifically the *Body to bar* indicator. These results do not allow us to decide whether the head or body orientation took priority if both sensors operated as desired. However, a deliberate manipulation can reveal repair strategies if sensors fail and thereby, provide insight into the structure and redundancies in human social signals. In this experiment, the available information was sufficient to the ghosts to identify and serve customers. Thus, a robot could rely on the body orientation only and would not require a high resolution camera and face tracking. For example, a mobile robot could save on energy by using cameras and trackers only when needed.

The color denotes whether the indicator attended less than (blue), equal to (green), or more than (red) expected by a random gaze distribution.

In addition to understanding the user’s behavior, the GiM paradigm allows us to determine which actions constitute a socially appropriate response. In the intention recognition trials, the ghosts had to communicate that they have noticed the customers and are ready to take their drink orders. Almost all ghosts decided to look at their customers, i.e., they visibly shifted the robot’s attention to the customer. This reflects the first part of a visual handshake. The customer can accept this invitation and complete the visual handshake by looking at the (robotic) bartender. The first part of offering a visual handshake and the second part of accepting it form an adjacency pair (Schegloff, 1968; Schegloff and Sacks, 1973) in a non-verbal modality. If completed, the handshake ensures that both sides are ready to begin a verbal communication. Argyle and Dean (1965) argued that mutual eye contact signals to both sides that the channel of verbal communication is open. Furthermore, establishing eye contact puts some pressure on the assistant to respond to the customer who has caught their eye (Goffman, 1963, p. 94). Vice-versa, avoiding eye contact is an effective method of avoiding a conversation in the first place (Goffman, 1963). However, looking at the customers could also be attributed to a visual inspection of the scene. But if the ghosts decided to look at something it was coherently the customer (96% of cases, see **Table 4**). Additionally, the dwell times provided evidence in favor of an intended action. First, the time spent on the control panel doubled if the ghosts acknowledged a customer. Secondly, the dwell times doubled on the addressed customer and reduced to one third for the other customer just before the ghosts initiated the handshake. Thirdly, 40% of the responses included a happy face that was directed toward the customer. This indicates that the ghosts invested additional efforts in a meaningful action rather than a casual visual inspection. Finally, the ghosts rarely selected actions other than a visual handshake. Only 19 times (12% of cases) a customer was prompted to place an order and only four times (3% of cases) a nodding head gesture was selected. In sum, a socially appropriate response to a new customer is to smilingly offer a visual handshake. The customer is then free to accept it by looking at the (robotic) bartender or to ignore it. This is very effective and at the same time less annoying than (repeatedly) inviting customers to place an order. Furthermore, this finding resembles observations in natural scenes and strengthens our conclusion that the GiM paradigm provides reliable insights. Thus, a robotic agent should employ this simple, effective but not annoying socially appropriate signal.

### Speech Recognition Trials

The speech recognition trials posed a greater challenge to the ghosts than intention recognition as evidenced by more and longer turns as well as longer dwell times on the panels (see **Tables 2** and **6**). We attributed this to the difficulty of interacting with pre-recorded customers and eliciting their orders. The pre-recorded nature of the customers also included the risk that the customers appear ignorant to the ghosts’ actions, specifically if they asked questions. However, the ghosts were as efficient as or better than the real robot and managed to serve a drink in 172 out of 174 interactions. This shows that (a) the ghosts performed well under challenging conditions, and (b) their responses can reveal useful strategies that improve interactions with service robots.

**FIGURE 4:**
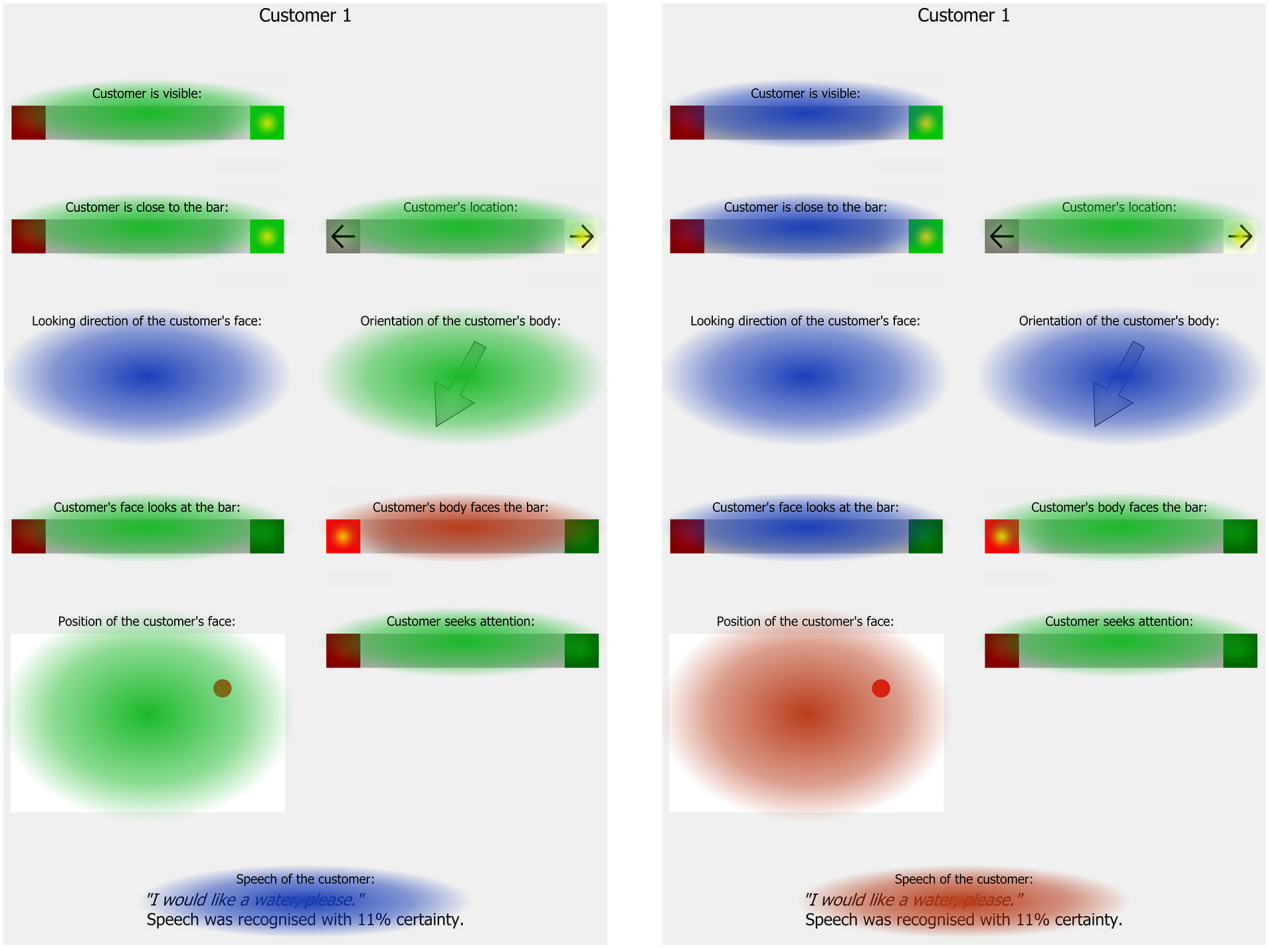
Comparison of relative dwell times on each indicator in the Response-turns in the intention recognition trials (left hand side) and the Serving-turns in the speech recognition trials (right hand side).

The analysis of the recognizer updates and the eye tracking data showed that once the interaction was initiated, the attention focus shifted from physical properties to the customer’s *Speech* (see **Figure 4**). For example, *Body to bar* was the most attended indicator in the intention recognition trials. In the intermediate speech recognition turns, its relative dwell time was reduced and reduced further during the *Serving*-turns such that it was not different from a random gaze pattern. At the same time, the dwell times on the *Speech* indicator increased. Thus, the closer the ghosts were to serving a drink, the more they shifted their attention away from physical properties in the visual sensory modality toward the customer’s speech in the verbal modality. As a result the customer’s speech was the single most attended indicator (see **Table 7**). As in the intention recognition trials, the ghosts subconsciously identified the most relevant modality from their social knowledge. In case of the orders, the social signal is essentially verbal and thus, the ghosts reduced their cognitive load by focussing on the *Speech*. The ghosts further reduced their load by focussing on the customer whom they would serve and spending significantly less time on the other customer especially when serving a drink (see **Table 6**). This adds converging evidence to our conclusion that the ghosts specifically scan for the expected social signals and thereby reduce their cognitive load.

Focussing on the socially relevant modality not only reduces the workload, it also prevents mistakes, e.g., abrupt terminations of the interaction. In one fifth of the servings the recognizers suggested that the customer was not close to the bar, in one third of the servings s/he was not visible, and her/his body was not oriented to the bar in half of the servings. In these cases, the ghosts would not have acknowledged a new customer but yet they served them a drink. In contrast, the robotic bartender at the evaluation assumed that customers must be visible and did not serve a drink. Instead, the robot terminated the interaction, waited until the customer was visible again and treated her/him as a new customer. Thus, the ghosts achieved a greater efficiency than the robot by continuing their interaction and serving the drink. We attribute this to the fact that the ghosts expected some closing to their conversation (Schegloff and Sacks, 1973), e.g., saying “Thank you”, rather than a sudden disappearance of the customer. Thus, the ghosts accepted the order even if the misleading data suggested the customer was (temporarily) not visible to the recognizers. In conclusion, a robot cannot expect to detect the customers as bidding for attention throughout the interaction. For example, if the customer moves or leans onto the bar, the recognizers can fail temporarily. However, the robot can expect some closing to the interaction and should not abruptly terminate the interaction as in the evaluation (Foster et al., 2012) and in a direction giving robot (Bohus et al., 2014). The robot still maintains its ability to detect whether the customer has actually left, e.g., if there is no speech input and the recognizers cannot detect the customer. Although it may be counter-intuitive to discard data, a smart weighting and ignoring some data can improve the robustness of a robotic agent and prevent abrupt terminations. In addition to improving the robot, focussing on the socially relevant modalities reflects cognitive principles in social interactions.

The ghosts strongly focussed on the *Speech* indicator. But another comparably large share of the relative dwell time was spent on the *Face position* indicator (see **Table 7**). Our analysis showed that this was partly due to the fact that the selector for serving a drink was spatially very close to the *Face position* of *Customer 2*. After accommodating for this confound, the ghosts attended the *Face position* reliably more than expected if their gaze randomly distributed across the information panel. It could be argued that the ghosts tried to establish eye contact to the customer by looking at a dot that depicted the customer’s face. This could be attributed to the fact that maintaining some level eye contact is important in a conversation because markedly looking away could signal that one is not an interested recipient (Schegloff and Sacks, 1973; Goodwin, 2000). However, maintaining eye contact would have been reasonable throughout the interaction and, importantly, whether or not the ghosts observed the dot was not visible to the customers. Thus, we cannot identify how the ghosts have particularly benefitted from the *Face position* indicator immediately before serving a drink (*Serving*-turns).

In the speech recognition trials, the ghosts predominantly tried to elicit which drink the customers ordered using verbal utterances in particular if the customer’s verbal utterance was unclear or recognized with a low confidence level. That means that the ghosts responded verbally to a verbal customer request. In contrast, the ghosts acknowledged a new customer by changing physical properties of the robot in the intention recognition trials, e.g., they manipulated the robot’s looking direction, but they did not speak. Thus, the ghosts preferred to respond in the same modality that was used by the customer. That means that the ghosts responded non-verbally to non-verbal actions and verbally to verbal actions. There was only one exception from this rule. If the ghosts served a drink, they responded with a physical action to a verbal request. However, this action was often accompanied by a verbal utterance (70% of the cases) and the customer specifically asked the bartender to serve a drink. In sum, the ghosts showed a strong preference to respond to a request within the same modality. Thus, a robotic agent should copy this human preference unless the user asked for a specific action.

The analysis of the ghosts’ responses after the customer placed an order revealed two strategies that contributed to their greater efficiency compared to the robotic bartender. As the robotic bartender, the ghosts decided in accordance with the confidence level of the ASR whether to serve the drink or to ask for a clarification. But their threshold for servings (*M*_serving_ = 59%, *M*_clarification_ = 29%) was lower than the 80% of the robotic bartender (Foster et al., 2012). Thus, firstly this threshold should be lowered to about 50% in order to serve the drink quicker. Secondly, the ghosts used echo questions in about half of their 123 clarification questions, i.e., they repeated the most likely guess of the ASR as a question (e.g., “A coke for you?”). A typical response would be a short confirmation (e.g., “Yes, please.”) or a correction (e.g., “No, I have ordered a juice.”). This strategy is particularly useful if the ASR has low confidence levels because the next challenge is to correctly identify the customer’s reply. Corrections tend to be delayed, prefaced, qualified and/or mitigated by an apology or an indirect form (Schegloff et al., 1977; Heritage, 1984). Thus, detecting whether the customer responded affirmative or with a correction could be achieved by simply analysing the length of the customer’s response. In this study, we used pre-recorded customer data. Thus, the customers could not respond to an echo question. But the data included the responses to the robot’s repeated prompts for an order (i.e., “What would you like?”). In turn, the ghosts perceived that their customers responded by repeating or slightly reformulating the original order with repeatedly low ASR confidence levels. Since the next turn after a question is typically perceived as a response (Schegloff, 1972; Sacks et al., 1974), a repetition such as “A coke, please. Confidence level 15%.” was perceived as more meaningful in the context of an echo question. Thus, the ghosts accepted the repetitions as positive answer and served the drink. Effectively, the ghosts retrospectively loaded their customer’s answer with an additional social meaning (Clark, 2012). But this strategy increased the redundancy in the interaction by repeating what has been said. However, this did not delay the serving but speeded the interaction and offered the customer to detect and correct communication errors. With a similar effect, the ghosts repeated the name of the drink in about half the servings (e.g., “Here is your coke.”) This allows the customer to silently accept this or to correct the robot in the last minute while the actions are already in preparation. In sum, the analysis of the confidence levels of ASR showed that about 50% is sufficient as a threshold. Furthermore, clarification requests and utterances accompanying (robot) actions such as servings should be formulated as echo questions or statements. Introducing redundancy by echoing essential information is socially appropriate and helps in achieving a smooth interaction, especially if the ASR confidence levels are low or in a noisy environment.

In addition to verbal utterances, the ghosts selected to look at the customer in most of their responses. This was the case if they asked for clarification, served a drink and even if they selected no other action. They maintained visual contact despite the fact that the control panel was reset after each update and required the ghosts to explicitly select this option in each response. Thus, we concluded that this option was important. Since the ghosts initiated the interaction by establishing visual contact to their customers, removing it could be interpreted as ending the interaction (Schegloff and Sacks, 1973; Goodwin, 2000). The ghosts almost constantly selected to look at their customers, but other options such as smiling and nodding were used more restrictedly. In particular, the ghosts nodded and smiled when confirming an order. Thus, they used these actions as an additional signal to confirm that the order was understood. As a result, confirming a drink order was a highly multimodal signal comprising of facial expressions, head gestures, verbal utterances and the serving itself. In this rich signal, the head gestures and facial expressions are redundant from a task-oriented perspective. However, they served the social purpose of clearly marking the serving to their customers. In sum, the ghosts maintained visual contact to their customers throughout the interaction. In contrast, nodding and smiling were used more restrictedly to confirm that the order was understood.

## Conclusion

The GiM paradigm is a reliable method for understanding the social behavior of users and the responses that they expect in a human–robot interaction setting. We demonstrated that results obtained with the GiM paradigm replicate findings that were obtained in the analyses of natural scenes, video recordings of natural scenes and in experiments using natural stimuli (Goffman, 1963; Argyle and Dean, 1965; Brouwer et al., 1979; Goodwin, 2000; Clark, 2012; Loth et al., 2013, 2015). In addition to experimenting with natural stimuli, the GiM paradigm allowed us to separately identify each single aspect of the scene (represented by a recognizer modality) that the ghost participants dedicated their overt attention to and its impact on their actions.

Our results showed that our ghost participants focussed on a small number of socially relevant modalities and ignored other, potentially misleading data. We argued that this is due to the ghosts scanning for particular social signals for recognizing the user’s intention and a general limitation of their cognitive resources (Broadbent, 1969; Allport et al., 1972; Mcleod, 1977). We also found that ignoring other data is advantageous as it hinders being distracted by misleading information that can lead to e.g., abrupt terminations of the interaction (Bohus et al., 2014). Thus, we demonstrated how fundamental principles of human cognition operate in social settings and also showed how a robotic agent can be improved by incorporating these principles.

Our study investigated two aspects of the bar setting: initiating the interaction, and ordering and serving the drink. The relevance of the modalities shifted as ghosts expected different social signals at each stage from their prior knowledge. When the customer tried to get the attention of the robotic bartender, her/his position and pose were most important. In contrast, the verbal modality was the most important for orders and servings. Thus, we have to identify which social signals are expected at each stage of an interaction and adapt the robotic policies to attend the relevant modalities. Furthermore, our findings showed that the ghosts preferred to respond in the same modality that the customer has used, i.e., changing the robot’s pose if the user signaled to them through their pose and position, and speaking if the user spoke to the robot. Thus, a multimodal grammar has to incorporate: (a) a method for focussing on the expected social signals and the relevant modalities, (b) keeping track of changes in expected signals and modalities, and (c) a preference to respond in the same modality as the user’s signal.

This GiM study revealed communication strategies that are simple, effective and socially appropriate. In acknowledgments, we showed that the robot should offer a visual handshake to the customers by looking at them and inviting them to join the interaction by looking at the robot, rather than annoy them by repeatedly inviting them to place an order. During the interaction, our ghost participants created redundancy by echoing salient parts of the customer’s utterance such as the drink order. Even though redundancy implies longer and more turns, this socially appropriate strategy required fewer turns and fewer clarification questions (cf. Giuliani et al., 2013) especially in cases involving inconclusive recognizer data. In sum, we found simple strategies for a smoother human–robot interaction that can enhance the robot’s multimodal grammar.

The ghost participants enjoyed the game-like interface of our GiM software and invested efforts and time into building a socially appropriate interaction with their customers. Thus, we concluded that our results reflect reliable, replicable insights in human social behavior and cognitive principles. Our initial study delivered substantial improvements for human–robot interaction policies by making the robot’s performance more robust, human-like and in turn, more predictable and enjoyable to its users. In our study, we used pre-recorded user data. But the GiM paradigm can be advanced into a real-time research tool in order to investigate the entire interaction. Furthermore, specific pieces of information or modalities can be manipulated in order to elicit repair and compensation strategies. The GiM interface can be adapted to various settings and its game-like experience makes it an ideal research tool for deriving multimodal grammars including strategies for recovering from inconclusive sensor data. Thus, the GiM paradigm is an effective, simple, and highly versatile method for understanding human social behavior that has the potential to revolutionize the field of social robotics.

## Conflict of Interest Statement

The authors declare that the research was conducted in the absence of any commercial or financial relationships that could be construed as a potential conflict of interest.

## Footnotes

1 In Psychology, the term modality refers to sensory modalities such as vision. In robotics, a modality tends to refer to a particular variable of the recognizer output. For example, the customer’s distance to the bar and her/his body orientation form two robotic modalities even though both are derived from the human visual modality. In order to avoid confusion, we distinguish sensory modality (e.g., vision) and recognizer modality (e.g., distance to the bar).

2 The other combinations showed the following results: *Face position* and *Body to bar R*^2^ = 0.303 with no statistically significant difference to the single predictor *Body to bar R*^2^ = 0.296, *Face position* and *Close to bar R*^2^ = 0.243 with no statistically significant difference to the single predictor *Close to bar R*^2^ = 0.244.

## References

[B1] Abelson R. P. (1981). Psychological status of the script concept. *Am. Psychol.* 36 715–729.

[B2] Abernethy B. Maxwell J. P. Jackson R. C. Masters R. S. W. (2007). “Skill in sport,” in *Handbook of Applied Cognition*, eds Durso F. T. Nickerson R. S. Dumais S. T. Lewandowsky S. Perfect T. J. (Chichester: John Wiley & Sons Ltd.), 333–359.

[B3] Admoni H. Dragan A. Srinivasa S. S. Scassellati B. (2014). “Deliberate delays during robot-to-human handovers improve compliance with gaze communication,” in *Proceedings of the 2014 ACM/IEEE International Conference on Human-robot Interaction* (Bielefeld: ACM Press), 49–56. 10.1145/2559636.2559682

[B4] Allport D. A. Antonis B. Reynolds P. (1972). On the division of attention: adisproof of the single channel hypothesis. *Q. J. Exp. Psychol.* 24 225–235. 10.1080/00335557243000102 5043119

[B5] Argyle M. Dean J. (1965). Eye-Contact, distance and affiliation. *Sociometry* 28 289–304. 10.2307/2786027 14341239

[B6] Baltzakis H. Pateraki M. Trahanias P. (2012). Visual tracking of hands, faces and facial features of multiple persons. *Mach. Vis. Appl.* 23 1141–1157. 10.1007/s00138-012-0409-5

[B7] Bohus D. Horvitz E. (2009a). “Dialog in the open world: platform and applications,” in *Proceedings of the 2009 International Conference on Multimodal Interfaces (ICMI-MLMI)* (Cambridge, MA: ACM Press), 31 10.1145/1647314.1647323

[B8] Bohus D. Horvitz E. (2009b). “Learning to predict engagement with a spoken dialog system in open-world settings,” in *Proceedings of the SIGDIAL 2009 Conference: The 10th Annual Meeting of the Special Interest Group on Discourse and Dialogue* (London: Association for Computational Linguistics), 244–252.

[B9] Bohus D. Horvitz E. (2009c). “Models for multiparty engagement in open-world dialog,” in *Proceedings of the SIGDIAL 2009 Conference: The 10th Annual Meeting of the Special Interest Group on Discourse and Dialogue* (London: Association for Computational Linguistics), 225–234.

[B10] Bohus D. Horvitz E. (2009d). “Open-world dialog: challenges, directions, and prototype,” in *Proceedings of the IJCAI’2009 Workshop on Knowledge and Reasoning in Practical Dialogue Systems*, Pasadena, CA.

[B11] Bohus D. Horvitz E. (2010). “On the challenges and opportunities of physically situated dialog,” in *Proceedings of the AAAI Fall Symposium Series*, Arlington, VA.

[B12] Bohus D. Horvitz E. (2011). “Multiparty turn taking in situated dialog: study, lessons, and directions,” in *Proceedings of the SIGDIAL 2011 Conference* (Portland, OR: Association for Computational Linguistics), 98–109.

[B13] Bohus D. Saw C. W. Horvitz E. (2014). “Directions robot: in-the-wild experiences and lessons learned,” in *Proceedings of the 13th International Conference on Autonomous Agents and Multiagent Systems* (Paris: International Foundation for Autonomous Agent and Multiagent Systems).

[B14] Breazeal C. DePalma N. Orkin J. Chernova S. Jung M. (2013). Crowdsourcing human-robot interaction: new methods and system evaluation in a public environment. *J. Human-Robot Interact.* 2 82–111. 10.5898/JHRI.2.1.Breazeal

[B15] Broadbent D. E. (1969). *Perception and Communication.* Oxford: Pergamon Press.

[B16] Brouwer D. Gerritsen M. De Haan D. (1979). Speech differences between women and men on the wrong track? *Lang. Soc.* 8 33–50. 10.1017/S0047404500005935

[B17] Clark H. H. (2003). “Pointing and placing,” in *Pointing?: Where Language, Culture, and Cognition Meet*, eds Kita S. (Mahwah, NJ: L. Erlbaum Associates), 243–268.

[B18] Clark H. H. (2012). “Wordless questions, wordless answers,” in *Questions: Formal, Functional and Interactional Perspectives*, ed. De Ruiter J. P. (Cambridge: Cambridge University Press), 81–102.

[B19] Dahlbäck N. Jönsson A. Ahrenberg L. (1993). “Wizard of Oz studies,” in *IUI ’93 Proceedings of the 1st international conference on Intelligent User Interfaces* (New York, NY: ACM Press), 193–200. 10.1145/169891.169968

[B20] Dalton P. Fraenkel N. (2012). Gorillas we have missed: sustained inattentional deafness for dynamic events. *Cognition* 124 367–372. 10.1016/j.cognition.2012.05.012 22726569

[B21] De Ruiter J. P. Cummins C. (2012). “A model of intentional communication: AIRBUS (Asymmetric Intention Recognition with Bayesian Updating of Signals),” *Presented at the Semantics and Pragmatics of Dialogue (SemDial)*, Paris.

[B22] faceLAB Eye Tracker (2009). *(Version 5). Tucson.* Arizona: Seeing Machines Inc.

[B23] Faul F. Erdfelder E. Lang A.-G. Buchner A. (2007). G*Power 3: a flexible statistical power analysis program for social, behavioral, and biomedical sciences. *Behav. Res. Methods* 39 175–191. 10.3758/BF03193146 17695343

[B24] Foster M. E. (2014). “Validating attention classifiers for multi-party human-robot interaction,” in *Proceedings of the 2014 ACM/IEEE International Conference on Human-Robot Interaction: Workshop on Attention Models in Robotics* (Bielefeld: ACM Press).

[B25] Foster M. E. Gaschler A. Giuliani M. (2013). “How can I help you? Comparing engagement classification strategies for a robot bartender,” in *Proceedings of the ACM International Conference on Multimodal Interaction (ICMI 2013)* (Sydney: ACM Press), 255–262. 10.1145/2522848.2522879

[B26] Foster M. E. Gaschler A. Giuliani M. Isard A. Pateraki M. Petrick R. P. A. (2012). “Two people walk into a bar: dynamic multi-party social interaction with a robot agent,” in *Proceedings of the 14th ACM International Conference on Multimodal Interaction (ICMI 2012)* (Santa Monica, CA: ACM Press). 10.1145/2388676.2388680

[B27] Fraser N. M. Gilbert G. N. (1991). Simulating speech systems. *Comput. Speech Lang.* 5 81–99. 10.1016/0885-2308(91)90019-M

[B28] Gaschler A. Huth K. Giuliani M. Kessler I. De Ruiter J. P. Knoll A. (2012). “Modelling state of interaction from head poses for social human-robot interaction,” in *Proceedings of the Gaze in Human- Robot Interaction Workshop held at the 7th ACM/IEEE International Conference on Human-Robot Interaction (HRI 2012)*, Boston.

[B29] Giuliani M. Petrick R. P. A. Foster M. E. Gaschler A. Isard A. Pateraki M. (2013). “Comparing task-based and socially intelligent behaviour in a robot bartender,” in *Proceedings of the 15th ACM on International Conference on Multimodal Interaction* (Sydney: ACM Press), 263–270. 10.1145/2522848.2522869

[B30] Goffman E. (1963). *Behaviour in Public Places.* Galt, ON: Collier-Macmillan

[B31] Goodrich M. A. Schultz A. C. (2007). Human-robot interaction: a survey. *Found. Trends Hum. Comput. Inter.* 1 203–275. 10.1561/1100000005

[B32] Goodwin C. (2000). Action and embodiment within situated human interaction. *J. Pragmat.* 32 1489–1522. 10.1016/S0378-2166(99)00096-X

[B33] Gray J. Breazeal C. Berlin M. Brooks A. Lieberman J. (2005). *Action Parsing and Goal Inference Using Self as Simulator.* Cambridge, MA: IEEE, 202–209. 10.1109/ROMAN.2005.1513780

[B34] Green A. Hüttenrauch H. Eklundh K. S. (2004). “Applying the wizard-of-Oz framework to cooperative service discovery and configuration,” in *RO-MAN 2004: 13th IEEE International Workshop on Robot and Human Interactive Communication: Proceedings: September 20-22 2004* (Kurashiki: IEEE).

[B35] Grice H. P. (1957). Meaning. *Philos. Rev.* 66:377 10.2307/2182440

[B36] Hall E. T. (1969). *The Hidden Dimension: An Anthropologist Examines Humans’ Use of Space in Public and Private.* New York, NY: Anchor Books, Doubleday & Company Inc.

[B37] Heritage J. (1984). *Garfinkel and Ethnomethodology.* New York, N.Y: Polity Press.

[B38] Holroyd A. Rich C. Sidner C. L. Ponsler B. (2011). “Generating connection events for human-robot collaboration,” in *Proceedings of the 20th IEEE International Symposium on Robot and Human Interactive Communication* (Atlanta, GA: IEEE), 241–246. 10.1109/ROMAN.2011.6005245

[B39] Java Runtime Environment (2012). *(Version 7). 500 Oracle Parkway.* Redwood Shores, CA: Oracle Corporation Available at: http://www.java.com

[B40] Jeannerod M. (2006). “Representations for actions,” in *Motor Cognition: What Actions Tell the Self*, eds D’Esposito M. Driver J. Robbins T. Schacter D. Treismann A. Weiskranz L. (Oxford, New York: Oxford University Press), 1–21.

[B41] Kelley J. F. (1984). An iterative design methodology for user-friendly natural language office information applications. *ACM Trans. Inf. Syst.* 2 26–41. 10.1145/357417.357420

[B42] Krämer N. Kopp S. Becker-Asano C. Sommer N. (2013). Smile and the world will smile with you—The effects of a virtual agent“s smile on users” evaluation and behavior. *Int. J. Hum. Comput. Stud.* 71 335–349. 10.1016/j.ijhcs.2012.09.006

[B43] Lakens D. Stel M. (2011). If they move in sync, they must feel in sync: movement synchrony leads to attributions of rapport and entitativity. *Soc. Cogn.* 29 1–14. 10.1521/soco.2011.29.1.1

[B44] Lee M. K. Forlizzi J. Rybski P. E. Crabbe F. Chung W. Finkle J. (2009). “The snackbot: documenting the design of a robot for long-term human-robot interaction,” in *Proceedings of the 4th ACM/IEEE International Conference on Human Robot Interaction* (La Jolla, CA: ACM Press), 7–14.

[B45] Levinson S. C. (1995). “Interactional biases in human thinking,” in *Social Intelligence and Interaction: Expressions and Implications of the Social Bias in Human Intelligence*, ed. Goody E. N. (New York, NY: Cambridge University Press), 221–260.

[B46] Lichtenthäler C. Peters A. Griffiths S. Kirsch A. (2013). “Social navigation - identifying robot navigation patterns in a path crossing scenario,” in *Social Robotics* Vol. 8239 eds Herrmann G. Pearson M. J. Lenz A. Bremner P. Spiers A. Leonards U. (Cham: Springer International Publishing), 84–93.

[B47] Liu X. Rieser V. Lemon O. (2009). “A wizard-of-oz interface to study information presentation strategies for spoken dialogue systems,” in *Proceedings of the First International Workshop on Spoken Dialogue Systems (IWSDS)*, Irsee.

[B48] Loth S. Giuliani M. De Ruiter J. P. (2014). “Ghost-in-the-machine: initial results,” in *Proceedings of the 2014 ACM/IEEE International Conference on Human-Robot Interaction*, Bielefeld, 234–235. 10.1145/2559636.2563696

[B49] Loth S. Huth K. De Ruiter J. P. (2013). Automatic detection of service initiation signals used in bars. *Front. Psychol.* 4:557 10.3389/fpsyg.2013.00557 PMC3757322 24009594

[B50] Loth S. Huth K. De Ruiter J. P. (2015). “Seeking attention: testing a model of initiating service Interactions,” in *A Multidisciplinary Approach to Service Encounters* Vol. 14 eds de la M. Hern ández-López O. Fernández Amaya L. (Amsterdam: Brill), 229–247.

[B51] Love J. Selker R. Verhagen J. Smira M. Wild A. Marsman M. (2014). *JASP (Version 0.5).* Amsterdam: JASP Available at: https://jasp-stats.org/

[B52] Mack A. Rock I. (1998). *Inattentional Blindness.* Cambridge, MA: MIT Press.

[B53] Mcleod P. (1977). A dual task response modality effect: support for multiprocessor models of attention. *Q. J. Exp. Psychol.* 29 651–667. 10.1080/14640747708400639

[B54] Michalowski M. P. Sabanovic S. Simmons R. (2006). “A spatial model of engagement for a social robot,” in *Proceedings of the 9th IEEE International Workshop on Advanced Motion Control* (Istanbul: IEEE), 762–767. 10.1109/AMC.2006.1631755

[B55] Morey R. D. Rouder J. N. Jamil T. (2014). *Package “BayesFactor” (Version 0.9.9) [R].* Groningen, NL: Rijksuniversiteit Groningen Available at: http://bayesfactorpcl.r-forge.r-project.org/

[B56] Néda Z. Ravasz E. Brechet Y. Vicsek T. Barabási A.-L. (2000). Self-organising processes: the sound of many hands clapping. *Nature* 403 849–850. 10.1038/35002660 10706271

[B57] Noël B. Furley P. van der Kamp J. Dicks M. Memmert D. (2014). The development of a method for identifying penalty kick strategies in association football. *J. Sports Sci.* 33 1–10. 10.1080/02640414.2014.926383 24914924

[B58] Orkin J. Roy D. (2007). The restaurant game: learning social behavior and language from thousands of players online. *J. Game Dev.* 3 39–60.

[B59] Orkin J. Roy D. (2009). “Automatic learning and generation of social behaviour from collective human gameplay,” in *Proceedings of the 8th International Conference on Autonomous Agents and Multimagent Systems?: May 10-15 2009* (Budapest: International Foundation for Autonomous Agent and Multiagent Systems).

[B60] Pateraki M. Baltzakis H. Trahanias P. (2014). Visual estimation of pointed targets for robot guidance via fusion of face pose and hand orientation. *Comput. Vis. Image Understand.* 120 1–13. 10.1016/j.cviu.2013.12.006

[B61] Petrick R. P. A. Foster M. E. (2012). “What would you like to drink? Recognising and planning with social states in a robot bartender domain,” in *Proceedings of the Twenty-Sixth AAAI Conference on Artificial Intelligence* (Toronto: AAAI Press), 69–76.

[B62] Petrick R. P. A. Foster M. E. (2013). “Plan-based social interaction with a robot bartender,” in *Proceedings of the ICAPS 2013 Application Showcase*, eds Policella N. Onder N. (Rome: ICAPS), 10–13.

[B63] Poppe R. (2010). A survey on vision-based human action recognition. *Image Vis. Comput.* 28 976–990. 10.1016/j.imavis.2009.11.014

[B64] R development core team (2007). *R: A Language And Environment For Statistical Computing (Version 2.12.0).* Wien: R Foundation for Statistical Computing.

[B65] Rich C. Ponsler B. Holroyd A. Sidner C. L. (2010). “Recognizing engagement in human-robot interaction,” in *Proceedings of the 5th ACM/IEEE International Conference on Human-Robot Interaction* (Osaka: ACM Press), 375–382. 10.1145/1734454.1734580

[B66] Richardson M. J. Marsh K. L. Isenhower R. W. Goodman J. R. L. Schmidt R. C. (2007). Rocking together: dynamics of intentional and unintentional interpersonal coordination. *Hum. Move. Sci.* 26 867–891. 10.1016/j.humov.2007.07.002 17765345

[B67] Riek L. (2012). Wizard of Oz studies in hri: a systematic review and new reporting guidelines. *J. Hum. Robot Inter.* 1 119–136. 10.5898/JHRI.1.1.Riek

[B68] Rieser V. Keizer S. Liu X. Lemon O. (2011). “Adaptive information presentation for spoken dialogue systems: evaluation with human subjects,” in *Proceedings of the 13th European Workshop on Natural Language Generation* (Nancy: Association for Computational Linguistics), 102–109.

[B69] Rieser V. Lemon O. (2009). Learning human multimodal dialogue strategies. *Nat. Lang. Eng.* 16 3–23. 10.1017/S1351324909005099

[B70] Ripley B. Venables W. (2014). *Package “nnet” (Version 7.3-8) [R].* Oxford, Oxforshire: University of Oxford Avilable at: http://www.stats.ox.ac.uk/pub/MASS4/

[B71] Rouder J. N. Speckman P. L. Sun D. Morey R. D. Iverson G. (2009). Bayesian t tests for accepting and rejecting the null hypothesis. *Psychon. Bull. Rev.* 16 225–237. 10.3758/PBR.16.2.225 19293088

[B72] Sacks H. Schegloff E. A. Jefferson G. (1974). A simplest systematics for the organization of turn-taking for conversation. *Language* 50 696–735. 10.2307/412243

[B73] Schank R. C. Abelson R. P. (1977). *Scripts, Plans, Goals and Understanding: An Inquiry Into Human Knowledge Structures.* Hillsdale, NJ: L. Erlbaum.

[B74] Schegloff E. A. (1968). Sequencing in conversational openings. *Am. Anthropol.* 70 1075–1095. 10.1525/aa.1968.70.6.02a00030

[B75] Schegloff E. A. (1972). “Notes on a conversational practice: formulating place,” in *Studies in Social Interaction*, ed. Sudnow D. (New York, NY: The Free Press and Collier-Macmillan Limited), 75–119.

[B76] Schegloff E. A. Jefferson G. Sacks H. (1977). The preference for self-correction in the organization of repair in conversation. *Language* 53 361–382. 10.2307/413107

[B77] Schegloff E. A. Sacks H. (1973). Opening up closings. *Semiotica* 8 289–327. 10.1515/semi.1973.8.4.289

[B78] Schorer J. Rienhoff R. Fischer L. Baker J. (2013). Foveal and peripheral fields of vision influences perceptual skill in anticipating opponents’ attacking position in volleyball. *Appl. Psychophysiol. Biofeedback* 38 185–192. 10.1007/s10484-013-9224-7 23775537

[B79] Shotton J. Sharp T. Kipman A. Fitzgibbon A. Finocchio M. Blake A. (2013). Real-time human pose recognition in parts from single depth images. *Commun. ACM* 56 116–124. 10.1145/2398356.2398381

[B80] Sidner C. L. Lee C. (2003). “Engagement rules for human-robot collaborative interactions,” in *IEEE International Conference on Systems, Man and Cybernetics*, Vol. 4 (Washington, DC: IEEE), 3957–3962. 10.1109/ICSMC.2003.1244506

[B81] Sidner C. L. Lee C. Kidd C. D. Lesh N. Rich C. (2005). Explorations in engagement for humans and robots. *Artif. Intell.* 166 140–164. 10.1016/j.artint.2005.03.005

[B82] Simons D. J. Chabris C. F. (1999). Gorillas in our midst: sustained inattentional blindness for dynamic events. *Perception* 28 1059–1074. 10.1068/p2952 10694957

[B83] Vinciarelli A. Pantic M. Heylen D. Pelachaud C. Poggi I. D’Errico F. (2012). Bridging the gap between social animal and unsocial machine: a survey of social signal processing. *IEEE Trans. Affect. Comput.* 3 69–87. 10.1109/T-AFFC.2011.27

[B84] von Ahn L. Dabbish L. (2008). Designing games with a purpose. *Commun. ACM* 51 58–67. 10.1145/1378704.1378719

[B85] Xu Y. Ohmoto Y. Ueda K. Komatsu T. Okadome T. Kamei K. (2010). Active adaptation in human-agent collaborative interaction. *J. Intell. Inf. Syst.* 37 23–38. 10.1007/s10844-010-0135-2

[B86] Yousuf A. M. Kobayashi Y. Yamazaki A. Yamazaki K. (2012). “Development of a mobile museum guide robot that can configure spatial formation with visitors,” in *Intelligent Computing Technology Berlin, Heidelberg* Vol. 7389 eds Huang D.-S. Jiang C. Bevilacqua V. Figueroa J. C. (Berlin: Springer), 432–432.

