# Supplementary material for: Ghost-in-the-Machine reveals human social signals for human–robot interaction
Source: Front Psychol. 2015 Nov 4;6:1641. doi: 10.3389/fpsyg.2015.01641 (PMC4631814; doi:10.3389/fpsyg.2015.01641)
Supplement: Supplementary file 1 [file Data_Sheet_1.DOCX]

Supplementary Material

Ghost-in-the-Machine Reveals Human Social Signals for Human-Robot Interaction

**Sebastian Loth^1*^, Katharina Jettka^1^, Manuel Giuliani^2^, Jan P. de Ruiter^1^**

^1^Psycholinguistics, Faculty for Linguistics and Literature, Bielefeld University, Germany

^2^Center for Human-Computer Interaction, University of Salzburg, Austria

*** Correspondence:** Sebastian Loth: Sebastian.Loth@uni-bielefeld.de

# Supplementary Data

The participants in the experiment observed data of pre-recorded customers (Foster et al., 2012). The examples below are taken from the log of the communication between the components of the robot and were used as input for the dedicated GiM software. The GiM software displayed this input on the user-interface as described in the main text. The log files below consist of several lines. Each line corresponds to one update in the GiM experiment. The order of presentation corresponded to the proper order according to the time stamp of the lines. The GiM software did not manipulate the data but only organised the presentation and recording of the responses.

The log files were filtered such that only two types of communication were included in the examples below and the experiment. First, the output of the social state estimator (Foster, Gaschler, & Giuliani, 2013) is listed as described in the main text. It comprises information about each agent in the scene labelled as A1 (agent 1) and A2 (agent 2). These correspond to Customer 1 and 2 in the GiM experiment. The agents are described by a number of variables, including their body orientation in degrees of angle, whether they seek attention (“!” means Boolean is false, “?” means value is unknown, no marking means Boolean is true), whether they are visible, left or right of centre and so on. Each indicator of the GiM interface corresponds to the respective variable in the output. These values are not associated to a confidence level. Secondly, the examples list the recognised speech from the Kinect speech recognition component. These lines include the utterance that has been recognized with its confidence level in brackets and the direction from where this utterance was detected in degrees of angle with its confidence level in brackets. The utterances were mapped to the respective customers based on whether they were to the left or to the right of the system (positive and negative value of the angle). The confidence level of the recognized speech was displayed in the experiment but the confidence level associated with the angle was not used.

## Example 1

-- 4/25/12 16:04:54:754 state: 1: Relations: [inTrans[]=nil, bodyOri[A1]=-33.75940295938743, !seeksAttention[A1], visible[A1], location[A1]=right, faceDir[A1]=-100000.0, !bodyLook[A1], !closeToBar[A1], !faceLook[A1], !faceSeen[A1], facePos[A1]=[-20.427838250885088, -161.25616311253162, -686.3799313377292], lastEvent[]=vision]

-- 4/25/12 16:04:57:900 state: 1: Relations: [inTrans[]=nil, bodyOri[A1]=2.006494487473111, seeksAttention[A1], visible[A1], location[A1]=right, faceDir[A1]=-100000.0, bodyLook[A1], closeToBar[A1], !faceLook[A1], !faceSeen[A1], facePos[A1]=[61.9424528235229, -389.2501873637751, -69.58958005168438], lastEvent[]=vision]

-- 4/25/12 16:05:00:113 state: 1: Relations: [inTrans[]=A1, bodyOri[A1]=-100000.0, seeksAttention[A1], visible[A1], location[A1]=left, faceDir[A1]=-100000.0, !bodyLook[A1], transHistory[robotAckAttention, A1], transHistory[robotGreeted, A1], closeToBar[A1], !faceLook[A1], !faceSeen[A1], facePos[A1]=[1693.3235178862287, 271.37443016704617, 1727.2539045653762], lastEvent[]=outputDone]

-- 4/25/12 16:05:02:117 state: 1: Relations: [inTrans[]=A1, bodyOri[A1]=-42.35912104634241, seeksAttention[A1], visible[A1], location[A1]=right, faceDir[A1]=-100000.0, !bodyLook[A1], transHistory[robotAckAttention, A1], transHistory[robotGreeted, A1], closeToBar[A1], !faceLook[A1], faceSeen[A1], facePos[A1]=[490.8987037719746, -209.7419093262069, 359.1327734583233], lastEvent[]=timeout]

-- 4/25/12 16:05:04:579 state: 1: Relations: [inTrans[]=A1, bodyOri[A1]=-100000.0, seeksAttention[A1], visible[A1], location[A1]=right, faceDir[A1]=-100000.0, !bodyLook[A1], transHistory[robotAckAttention, A1], transHistory[robotGreeted, A1], closeToBar[A1], !faceLook[A1], !faceSeen[A1], facePos[A1]=[899.824484542649, -11.350269169173004, 771.9359517668474], lastEvent[]=outputDone]

-- 4/25/12 16:05:04:583 kinect-speech: 1: recognized: Dankeschön (0,25317907333374) 11,6568409022208 (0,39773017628865)

-- 4/25/12 16:05:06:073 kinect-speech: 1: recognized: Okay (0,358727335929871) -6,01792469734929 (1)

-- 4/25/12 16:05:07:524 kinect-speech: 1: recognized: eine Cola (0,543479263782501) -7,57497969329579 (0,501914885038643)

-- 4/25/12 16:05:07:594 state: 1: Relations: [inTrans[]=A1, bodyOri[A1]=-100000.0, !badASR[A1], visible[A1], faceDir[A1]=-100000.0, location[A1]=right, closeToBar[A1], transHistory[robotAckAttention, A1], transHistory[robotGreeted, A1], transHistory[agentOrdered, A1], facePos[A1]=[467.67589704367936, -173.12446625778364, 319.3465316423933], seeksAttention[A1], !bodyLook[A1], request[A1]=coke, !faceLook[A1], faceSeen[A1], lastEvent[]=userSpeech]

-- 4/25/12 16:05:10:069 kinect-speech: 1: recognized: Dankeschön (0,312276810407639) -8,06703848181441 (0,678347759282884)

-- 4/25/12 16:05:10:127 state: 1: Relations: [inTrans[]=A1, bodyOri[A1]=-100000.0, !badASR[A1], visible[A1], faceDir[A1]=-100000.0, location[A1]=right, closeToBar[A1], transHistory[robotAckAttention, A1], transHistory[robotGreeted, A1], transHistory[agentOrdered, A1], transHistory[robotServed, A1], facePos[A1]=[559.9051214025718, -138.06814434398345, 437.7350530253692], seeksAttention[A1], !bodyLook[A1], request[A1]=coke, !faceLook[A1], !faceSeen[A1], lastEvent[]=outputDone]

-- 4/25/12 16:05:12:078 kinect-speech: 1: recognized: eine Cola (0,235002711415291) -7,03940221940577 (1)

-- 4/25/12 16:05:15:130 state: 1: Relations: [inTrans[]=A1, bodyOri[A1]=-100000.0, !badASR[A1], visible[A1], faceDir[A1]=-100000.0, location[A1]=right, closeToBar[A1], transHistory[robotAckAttention, A1], transHistory[robotGreeted, A1], transHistory[agentOrdered, A1], transHistory[robotServed, A1], facePos[A1]=[761.0573410776095, -78.72235699386559, 635.50377582438], seeksAttention[A1], !bodyLook[A1], request[A1]=coke, !faceLook[A1], !faceSeen[A1], lastEvent[]=timeout]

## Example 2

-- 4/26/12 11:10:22:749 kinect-speech: 1: recognized: Tschüss (0,00344359874725342) 2,45971479149247 (0,386005730182847)

-- 4/26/12 11:10:24:575 kinect-speech: 1: recognized: Nein (0,0838528275489807) 1,21763031304319 (0,328244627753669)

-- 4/26/12 11:10:30:266 state: 1: Relations: [inTrans[]=nil, bodyOri[A1]=-27.175903354394443, ?seeksAttention[A1], visible[A1], location[A1]=left, faceDir[A1]=-100000.0, !bodyLook[A1], closeToBar[A1], !faceLook[A1], !faceSeen[A1], facePos[A1]=[51.8761859189126, 633.5082024895041, -171.00327806307473], lastEvent[]=vision]

-- 4/26/12 11:10:30:535 state: 1: Relations: [inTrans[]=nil, bodyOri[A1]=-1.2159509997777624, ?seeksAttention[A1], visible[A1], location[A1]=left, faceDir[A1]=-100000.0, bodyLook[A1], closeToBar[A1], !faceLook[A1], !faceSeen[A1], facePos[A1]=[65.44681122908582, 584.6834704380065, -70.50871698006063], lastEvent[]=vision]

-- 4/26/12 11:10:32:476 state: 1: Relations: [inTrans[]=A1, bodyOri[A1]=62.20374835162733, ?seeksAttention[A1], visible[A1], location[A1]=left, faceDir[A1]=-100000.0, !bodyLook[A1], transHistory[robotAckAttention, A1], transHistory[robotGreeted, A1], closeToBar[A1], !faceLook[A1], !faceSeen[A1], facePos[A1]=[14.705494010108168, 608.5962647852457, -81.84556266045661], lastEvent[]=outputDone]

-- 4/26/12 11:10:32:518 state: 1: Relations: [inTrans[]=A1, bodyOri[A1]=54.98738522272601, ?seeksAttention[A1], visible[A1], location[A1]=left, faceDir[A1]=-100000.0, !bodyLook[A1], transHistory[robotAckAttention, A1], transHistory[robotGreeted, A1], closeToBar[A1], !faceLook[A1], !faceSeen[A1], facePos[A1]=[13.116096213270112, 608.9894575097227, -82.14076332642912], bodyOri[A2]=37.540882948031374, ?seeksAttention[A2], visible[A2], location[A2]=left, faceDir[A2]=-100000.0, !bodyLook[A2], !closeToBar[A2], !faceLook[A2], faceSeen[A2], facePos[A2]=[263.70538477745845, 38.5432257461226, -795.1088682696284], lastEvent[]=vision]

-- 4/26/12 11:10:33:575 state: 1: Relations: [inTrans[]=A1, bodyOri[A1]=32.0537549772773, ?seeksAttention[A1], visible[A1], location[A1]=left, faceDir[A1]=-100000.0, !bodyLook[A1], transHistory[robotAckAttention, A1], transHistory[robotGreeted, A1], closeToBar[A1], !faceLook[A1], !faceSeen[A1], facePos[A1]=[8.437952408116189, 609.1839060545792, -80.0172968193674], bodyOri[A2]=-3.747013984106482, ?seeksAttention[A2], visible[A2], location[A2]=right, faceDir[A2]=-100000.0, bodyLook[A2], closeToBar[A2], !faceLook[A2], faceSeen[A2], facePos[A2]=[281.79455357890765, -296.913677159492, -292.50457017710755], lastEvent[]=vision]

-- 4/26/12 11:10:34:987 state: 1: Relations: [inTrans[]=A1, bodyOri[A1]=-11.583843947974726, ?seeksAttention[A1], visible[A1], location[A1]=left, faceDir[A1]=-100000.0, !bodyLook[A1], transHistory[robotAckAttention, A1], transHistory[robotGreeted, A1], closeToBar[A1], !faceLook[A1], !faceSeen[A1], facePos[A1]=[1.3756208788843196, 609.8071199025117, -88.17103971510119], bodyOri[A2]=-6.480840756187899, ?seeksAttention[A2], visible[A2], location[A2]=right, faceDir[A2]=-100000.0, bodyLook[A2], closeToBar[A2], !faceLook[A2], faceSeen[A2], facePos[A2]=[287.3819382288318, -277.3744088901404, -65.69398983257884], lastEvent[]=outputDone]

-- 4/26/12 11:10:35:943 state: 1: Relations: [inTrans[]=A1, ?seeksAttention[A1], !visible[A1], location[A1]=left, !bodyLook[A1], transHistory[robotAckAttention, A1], transHistory[robotGreeted, A1], closeToBar[A1], !faceLook[A1], !faceSeen[A1], bodyOri[A2]=-100000.0, ?seeksAttention[A2], visible[A2], location[A2]=right, faceDir[A2]=-100000.0, !bodyLook[A2], closeToBar[A2], !faceLook[A2], faceSeen[A2], facePos[A2]=[283.57403173117405, -271.69581296623466, -46.66743744853693], lastEvent[]=vision]

-- 4/26/12 11:10:38:395 kinect-speech: 1: recognized: Einen Saft bitte. (0,281915664672852) 13,3450360536796 (0,572783485588801)

-- 4/26/12 11:10:38:488 state: 1: Relations: [inTrans[]=A1, bodyOri[A1]=-100000.0, ?seeksAttention[A1], visible[A1], faceDir[A1]=-100000.0, location[A1]=left, !bodyLook[A1], transHistory[robotAckAttention, A1], transHistory[robotGreeted, A1], closeToBar[A1], !faceLook[A1], facePos[A1]=[686.856514047518, 315.39432362884133, 629.5702265150175], !faceSeen[A1], bodyOri[A2]=-21.4762487815766, ?seeksAttention[A2], visible[A2], location[A2]=right, faceDir[A2]=-100000.0, !bodyLook[A2], transHistory[robotAckAttention, A2], transHistory[robotInitWait, A2], closeToBar[A2], !faceLook[A2], faceSeen[A2], facePos[A2]=[270.5183722132457, -277.2513915510068, -59.901724532091976], lastEvent[]=outputDone]

-- 4/26/12 11:10:40:495 state: 1: Relations: [inTrans[]=A1, bodyOri[A1]=-100000.0, ?seeksAttention[A1], visible[A1], faceDir[A1]=-100000.0, location[A1]=left, !bodyLook[A1], transHistory[robotAckAttention, A1], transHistory[robotGreeted, A1], closeToBar[A1], !faceLook[A1], facePos[A1]=[773.9117846428733, 279.1854539603656, 715.0256100813789], !faceSeen[A1], bodyOri[A2]=-9.63775565945233, ?seeksAttention[A2], visible[A2], location[A2]=right, faceDir[A2]=-100000.0, bodyLook[A2], transHistory[robotAckAttention, A2], transHistory[robotInitWait, A2], closeToBar[A2], !faceLook[A2], faceSeen[A2], facePos[A2]=[269.63449464252085, -279.7986815668485, -62.01936748824005], lastEvent[]=timeout]

-- 4/26/12 11:10:42:830 state: 1: Relations: [inTrans[]=A1, bodyOri[A1]=-100000.0, ?seeksAttention[A1], visible[A1], faceDir[A1]=-100000.0, location[A1]=left, !bodyLook[A1], transHistory[robotAckAttention, A1], transHistory[robotGreeted, A1], closeToBar[A1], !faceLook[A1], facePos[A1]=[842.1712473677151, 250.77759404863696, 783.4105856394966], !faceSeen[A1], bodyOri[A2]=-100000.0, ?seeksAttention[A2], visible[A2], location[A2]=right, faceDir[A2]=-100000.0, !bodyLook[A2], transHistory[robotAckAttention, A2], transHistory[robotInitWait, A2], closeToBar[A2], !faceLook[A2], faceSeen[A2], facePos[A2]=[279.24588507675116, -270.3422713241906, -43.580248464996885], lastEvent[]=outputDone]

-- 4/26/12 11:10:43:333 kinect-speech: 1: recognized: Dankeschön (0,0825322568416595) 10,9093900713086 (0,34465422395733)

-- 4/26/12 11:10:44:444 kinect-speech: 1: recognized: Eine Cola bitte. (0,882581353187561) 12,653376734383 (0,332851728383978)

-- 4/26/12 11:10:44:587 state: 1: Relations: [inTrans[]=A1, bodyOri[A1]=-100000.0, !badASR[A1], visible[A1], location[A1]=left, faceDir[A1]=-100000.0, closeToBar[A1], transHistory[robotAckAttention, A1], transHistory[robotGreeted, A1], transHistory[agentOrdered, A1], facePos[A1]=[756.6892952683486, 281.1919830494499, 704.2754276392961], ?seeksAttention[A1], !bodyLook[A1], request[A1]=coke, !faceLook[A1], !faceSeen[A1], bodyOri[A2]=68.24255561005776, ?seeksAttention[A2], visible[A2], location[A2]=right, faceDir[A2]=-100000.0, !bodyLook[A2], transHistory[robotAckAttention, A2], transHistory[robotInitWait, A2], closeToBar[A2], !faceLook[A2], faceSeen[A2], facePos[A2]=[271.57150482084364, -275.89195189473685, -53.919269594668094], lastEvent[]=userSpeech]

-- 4/26/12 11:10:45:853 state: 1: Relations: [inTrans[]=A1, !badASR[A1], !visible[A1], location[A1]=left, closeToBar[A1], transHistory[robotAckAttention, A1], transHistory[robotGreeted, A1], transHistory[agentOrdered, A1], ?seeksAttention[A1], !bodyLook[A1], request[A1]=coke, !faceLook[A1], faceSeen[A1], bodyOri[A2]=82.11993119170276, ?seeksAttention[A2], visible[A2], location[A2]=right, faceDir[A2]=-100000.0, !bodyLook[A2], transHistory[robotAckAttention, A2], transHistory[robotInitWait, A2], closeToBar[A2], !faceLook[A2], faceSeen[A2], facePos[A2]=[278.0700061024352, -278.56572111075724, -49.96867709980074], lastEvent[]=vision]

-- 4/26/12 11:10:47:287 state: 1: Relations: [inTrans[]=A1, !badASR[A1], !visible[A1], location[A1]=left, closeToBar[A1], transHistory[robotAckAttention, A1], transHistory[robotGreeted, A1], transHistory[agentOrdered, A1], transHistory[robotServed, A1], ?seeksAttention[A1], !bodyLook[A1], request[A1]=coke, !faceLook[A1], faceSeen[A1], bodyOri[A2]=-18.115372460125265, ?seeksAttention[A2], visible[A2], location[A2]=right, faceDir[A2]=-100000.0, !bodyLook[A2], transHistory[robotAckAttention, A2], transHistory[robotInitWait, A2], closeToBar[A2], !faceLook[A2], faceSeen[A2], facePos[A2]=[269.02191920492464, -267.66148018619543, -40.37156932641278], lastEvent[]=outputDone]

-- 4/26/12 11:10:52:290 state: 1: Relations: [inTrans[]=A1, bodyOri[A1]=10.619239187369967, !badASR[A1], visible[A1], faceDir[A1]=-100000.0, location[A1]=left, closeToBar[A1], transHistory[robotAckAttention, A1], transHistory[robotGreeted, A1], transHistory[agentOrdered, A1], transHistory[robotServed, A1], facePos[A1]=[-6.206971627320627, 608.114014521623, -54.35917826834657], ?seeksAttention[A1], !bodyLook[A1], request[A1]=coke, !faceLook[A1], !faceSeen[A1], bodyOri[A2]=-100000.0, ?seeksAttention[A2], visible[A2], location[A2]=right, faceDir[A2]=-100000.0, !bodyLook[A2], transHistory[robotAckAttention, A2], transHistory[robotInitWait, A2], closeToBar[A2], !faceLook[A2], faceSeen[A2], facePos[A2]=[250.0079789863969, -262.634058198383, -39.93486631619703], lastEvent[]=timeout]

-- 4/26/12 11:11:04:506 state: 1: Relations: [inTrans[]=A1, !badASR[A1], !visible[A1], location[A1]=left, closeToBar[A1], transHistory[robotAckAttention, A1], transHistory[robotGreeted, A1], transHistory[agentOrdered, A1], transHistory[robotServed, A1], ?seeksAttention[A1], !bodyLook[A1], request[A1]=coke, !faceLook[A1], faceSeen[A1], bodyOri[A2]=-10.080709498781486, ?seeksAttention[A2], visible[A2], location[A2]=right, faceDir[A2]=-100000.0, !bodyLook[A2], transHistory[robotAckAttention, A2], transHistory[robotInitWait, A2], closeToBar[A2], !faceLook[A2], faceSeen[A2], facePos[A2]=[282.08107198136463, -261.8724627458353, -26.054819190603894], lastEvent[]=vision]

-- 4/26/12 11:11:08:207 state: 1: Relations: [inTrans[]=nil, bodyOri[A1]=-100000.0, !badASR[A1], visible[A1], faceDir[A1]=-100000.0, location[A1]=left, closeToBar[A1], transHistory[robotAckAttention, A1], transHistory[robotGreeted, A1], transHistory[agentOrdered, A1], transHistory[robotServed, A1], transHistory[transComplete, A1], facePos[A1]=[216.4267401897407, 959.1004369116554, -90.56278987190058], ?seeksAttention[A1], !bodyLook[A1], request[A1]=coke, !faceLook[A1], faceSeen[A1], bodyOri[A2]=-16.32939910376009, ?seeksAttention[A2], visible[A2], location[A2]=right, faceDir[A2]=-100000.0, !bodyLook[A2], transHistory[robotAckAttention, A2], transHistory[robotInitWait, A2], closeToBar[A2], !faceLook[A2], faceSeen[A2], facePos[A2]=[279.27202569536166, -269.1882889451255, -32.69277986121233], lastEvent[]=outputDone]

-- 4/26/12 11:11:08:215 state: 1: Relations: [inTrans[]=nil, !badASR[A1], !visible[A1], location[A1]=left, closeToBar[A1], transHistory[robotAckAttention, A1], transHistory[robotGreeted, A1], transHistory[agentOrdered, A1], transHistory[robotServed, A1], transHistory[transComplete, A1], ?seeksAttention[A1], !bodyLook[A1], request[A1]=coke, !faceLook[A1], faceSeen[A1], bodyOri[A2]=-18.03619760396719, ?seeksAttention[A2], visible[A2], location[A2]=right, faceDir[A2]=-100000.0, !bodyLook[A2], transHistory[robotAckAttention, A2], transHistory[robotInitWait, A2], closeToBar[A2], !faceLook[A2], faceSeen[A2], facePos[A2]=[277.3308549041094, -269.48136826804915, -34.42215889993054], lastEvent[]=vision]

-- 4/26/12 11:11:09:130 state: 1: Relations: [inTrans[]=A2, !badASR[A1], !visible[A1], location[A1]=left, closeToBar[A1], transHistory[robotAckAttention, A1], transHistory[robotGreeted, A1], transHistory[agentOrdered, A1], transHistory[robotServed, A1], transHistory[transComplete, A1], ?seeksAttention[A1], !bodyLook[A1], request[A1]=coke, !faceLook[A1], faceSeen[A1], bodyOri[A2]=-29.66793766341228, ?seeksAttention[A2], visible[A2], location[A2]=right, faceDir[A2]=-100000.0, !bodyLook[A2], transHistory[robotAckAttention, A2], transHistory[robotGreeted, A2], closeToBar[A2], !faceLook[A2], faceSeen[A2], facePos[A2]=[269.8435082009337, -270.92311946339174, -41.593320697918216], lastEvent[]=outputDone]

-- 4/26/12 11:11:09:558 kinect-speech: 1: recognized: Tschüss (0,00523450970649719) 1,68541091861772 (0,42538334025899)

-- 4/26/12 11:11:11:002 state: 1: Relations: [inTrans[]=A2, bodyOri[A1]=-100000.0, !badASR[A1], visible[A1], faceDir[A1]=-100000.0, location[A1]=left, closeToBar[A1], transHistory[robotAckAttention, A1], transHistory[robotGreeted, A1], transHistory[agentOrdered, A1], transHistory[robotServed, A1], transHistory[transComplete, A1], facePos[A1]=[68.42998763474543, 1028.7090018101596, -253.38948597237845], ?seeksAttention[A1], !bodyLook[A1], request[A1]=coke, !faceLook[A1], !faceSeen[A1], bodyOri[A2]=-18.466527810738064, ?seeksAttention[A2], visible[A2], location[A2]=right, faceDir[A2]=-100000.0, !bodyLook[A2], transHistory[robotAckAttention, A2], transHistory[robotGreeted, A2], closeToBar[A2], !faceLook[A2], faceSeen[A2], facePos[A2]=[265.74745129177245, -278.8995101264301, -50.45813910316042], lastEvent[]=outputDone]

-- 4/26/12 11:11:11:085 kinect-speech: 1: recognized: a coke please (0,314753651618958) 9,99018806193885 (0,345700793584524)

-- 4/26/12 11:11:13:005 state: 1: Relations: [inTrans[]=A2, bodyOri[A1]=-100000.0, !badASR[A1], visible[A1], faceDir[A1]=-100000.0, location[A1]=left, !closeToBar[A1], transHistory[robotAckAttention, A1], transHistory[robotGreeted, A1], transHistory[agentOrdered, A1], transHistory[robotServed, A1], transHistory[transComplete, A1], facePos[A1]=[-176.89975231008634, 1222.5116056138404, -526.5477338534386], ?seeksAttention[A1], !bodyLook[A1], request[A1]=coke, !faceLook[A1], !faceSeen[A1], bodyOri[A2]=-12.132336540452602, ?seeksAttention[A2], visible[A2], location[A2]=right, faceDir[A2]=-100000.0, !bodyLook[A2], transHistory[robotAckAttention, A2], transHistory[robotGreeted, A2], closeToBar[A2], !faceLook[A2], faceSeen[A2], facePos[A2]=[251.23243576509162, -269.2006389656237, 29.36207557070361], lastEvent[]=timeout]

-- 4/26/12 11:11:13:293 state: 1: Relations: [inTrans[]=A2, !badASR[A1], !visible[A1], location[A1]=left, !closeToBar[A1], transHistory[robotAckAttention, A1], transHistory[robotGreeted, A1], transHistory[agentOrdered, A1], transHistory[robotServed, A1], transHistory[transComplete, A1], ?seeksAttention[A1], !bodyLook[A1], request[A1]=coke, !faceLook[A1], !faceSeen[A1], bodyOri[A2]=-12.022028353948071, ?seeksAttention[A2], visible[A2], location[A2]=right, faceDir[A2]=-100000.0, !bodyLook[A2], transHistory[robotAckAttention, A2], transHistory[robotGreeted, A2], closeToBar[A2], !faceLook[A2], faceSeen[A2], facePos[A2]=[259.78776833636266, -264.3813279644921, 30.373588236523346], lastEvent[]=vision]

-- 4/26/12 11:11:15:345 state: 1: Relations: [inTrans[]=A2, !badASR[A1], !visible[A1], location[A1]=left, !closeToBar[A1], transHistory[robotAckAttention, A1], transHistory[robotGreeted, A1], transHistory[agentOrdered, A1], transHistory[robotServed, A1], transHistory[transComplete, A1], ?seeksAttention[A1], !bodyLook[A1], request[A1]=coke, !faceLook[A1], !faceSeen[A1], bodyOri[A2]=-29.883971660903008, ?seeksAttention[A2], visible[A2], location[A2]=right, faceDir[A2]=-100000.0, !bodyLook[A2], transHistory[robotAckAttention, A2], transHistory[robotGreeted, A2], closeToBar[A2], !faceLook[A2], faceSeen[A2], facePos[A2]=[259.50271401965915, -270.7082528449195, 19.811440174686368], lastEvent[]=outputDone]

-- 4/26/12 11:11:15:459 kinect-speech: 1: recognized: Ich möchte bitte einen Saft. (0,685819685459137) 12,6816700889885 (0,423772867085129)

-- 4/26/12 11:11:17:617 kinect-speech: 1: recognized: a juice (0,938063740730286) -5,7520948608188 (1)

-- 4/26/12 11:11:17:636 state: 1: Relations: [inTrans[]=A2, !badASR[A1], !visible[A1], location[A1]=left, !closeToBar[A1], transHistory[robotAckAttention, A1], transHistory[robotGreeted, A1], transHistory[agentOrdered, A1], transHistory[robotServed, A1], transHistory[transComplete, A1], ?seeksAttention[A1], !bodyLook[A1], request[A1]=coke, !faceLook[A1], !faceSeen[A1], bodyOri[A2]=-100000.0, !badASR[A2], visible[A2], faceDir[A2]=-100000.0, location[A2]=right, closeToBar[A2], transHistory[robotAckAttention, A2], transHistory[robotGreeted, A2], transHistory[agentOrdered, A2], facePos[A2]=[258.22570474306417, -269.19146508324394, 18.534254928074006], ?seeksAttention[A2], !bodyLook[A2], request[A2]=juice, !faceLook[A2], faceSeen[A2], lastEvent[]=userSpeech]

-- 4/26/12 11:11:20:378 state: 1: Relations: [inTrans[]=A2, bodyOri[A1]=-100000.0, !badASR[A1], visible[A1], faceDir[A1]=-100000.0, location[A1]=left, closeToBar[A1], transHistory[robotAckAttention, A1], transHistory[robotGreeted, A1], transHistory[agentOrdered, A1], transHistory[robotServed, A1], transHistory[transComplete, A1], facePos[A1]=[597.1576856674974, 560.6071751190175, 462.2304771025151], ?seeksAttention[A1], !bodyLook[A1], request[A1]=coke, !faceLook[A1], !faceSeen[A1], bodyOri[A2]=-100000.0, !badASR[A2], visible[A2], faceDir[A2]=-100000.0, location[A2]=right, closeToBar[A2], transHistory[robotAckAttention, A2], transHistory[robotGreeted, A2], transHistory[agentOrdered, A2], transHistory[robotServed, A2], facePos[A2]=[230.46347816171522, -254.7726036141775, 21.36491480038285], ?seeksAttention[A2], !bodyLook[A2], request[A2]=juice, !faceLook[A2], faceSeen[A2], lastEvent[]=outputDone]

-- 4/26/12 11:11:20:493 kinect-speech: 1: recognized: Dankeschön (0,039694607257843) 10,9996584007197 (0,470472384019854)

-- 4/26/12 11:11:21:425 state: 1: Relations: [inTrans[]=A2, !badASR[A1], !visible[A1], location[A1]=left, closeToBar[A1], transHistory[robotAckAttention, A1], transHistory[robotGreeted, A1], transHistory[agentOrdered, A1], transHistory[robotServed, A1], transHistory[transComplete, A1], ?seeksAttention[A1], !bodyLook[A1], request[A1]=coke, !faceLook[A1], !faceSeen[A1], bodyOri[A2]=-100000.0, !badASR[A2], visible[A2], faceDir[A2]=-100000.0, location[A2]=right, closeToBar[A2], transHistory[robotAckAttention, A2], transHistory[robotGreeted, A2], transHistory[agentOrdered, A2], transHistory[robotServed, A2], facePos[A2]=[228.01952432998382, -256.1573049241485, 19.223286877171404], ?seeksAttention[A2], !bodyLook[A2], request[A2]=juice, !faceLook[A2], faceSeen[A2], lastEvent[]=vision]

-- 4/26/12 11:11:24:393 state: 1: Relations: [inTrans[]=A2, !badASR[A1], !visible[A1], location[A1]=left, closeToBar[A1], transHistory[robotAckAttention, A1], transHistory[robotGreeted, A1], transHistory[agentOrdered, A1], transHistory[robotServed, A1], transHistory[transComplete, A1], ?seeksAttention[A1], !bodyLook[A1], request[A1]=coke, !faceLook[A1], !faceSeen[A1], bodyOri[A2]=-100000.0, !badASR[A2], visible[A2], faceDir[A2]=-100000.0, location[A2]=right, closeToBar[A2], transHistory[robotAckAttention, A2], transHistory[robotGreeted, A2], transHistory[agentOrdered, A2], transHistory[robotServed, A2], facePos[A2]=[222.43112125026073, -255.4267205597365, 23.84903550442482], ?seeksAttention[A2], !bodyLook[A2], request[A2]=juice, !faceLook[A2], faceSeen[A2], lastEvent[]=vision]

-- 4/26/12 11:11:27:692 state: 1: Relations: [inTrans[]=A2, !badASR[A1], !visible[A1], location[A1]=left, closeToBar[A1], transHistory[robotAckAttention, A1], transHistory[robotGreeted, A1], transHistory[agentOrdered, A1], transHistory[robotServed, A1], transHistory[transComplete, A1], ?seeksAttention[A1], !bodyLook[A1], request[A1]=coke, !faceLook[A1], faceSeen[A1], bodyOri[A2]=-41.57485333764966, !badASR[A2], visible[A2], faceDir[A2]=-100000.0, location[A2]=right, closeToBar[A2], transHistory[robotAckAttention, A2], transHistory[robotGreeted, A2], transHistory[agentOrdered, A2], transHistory[robotServed, A2], facePos[A2]=[226.55966061456047, -257.7160828332674, 21.135191096589324], ?seeksAttention[A2], !bodyLook[A2], request[A2]=juice, !faceLook[A2], faceSeen[A2], lastEvent[]=vision]

-- 4/26/12 11:11:31:741 state: 1: Relations: [inTrans[]=A2, !badASR[A1], !visible[A1], location[A1]=left, closeToBar[A1], transHistory[robotAckAttention, A1], transHistory[robotGreeted, A1], transHistory[agentOrdered, A1], transHistory[robotServed, A1], transHistory[transComplete, A1], ?seeksAttention[A1], !bodyLook[A1], request[A1]=coke, !faceLook[A1], !faceSeen[A1], bodyOri[A2]=-19.4143676912881, !badASR[A2], visible[A2], faceDir[A2]=-100000.0, location[A2]=right, closeToBar[A2], transHistory[robotAckAttention, A2], transHistory[robotGreeted, A2], transHistory[agentOrdered, A2], transHistory[robotServed, A2], facePos[A2]=[222.86984914738116, -247.45129572435957, -8.853647535737991], ?seeksAttention[A2], !bodyLook[A2], request[A2]=juice, !faceLook[A2], faceSeen[A2], lastEvent[]=vision]

-- 4/26/12 11:11:41:306 state: 1: Relations: [inTrans[]=nil, bodyOri[A1]=-100000.0, !badASR[A1], visible[A1], faceDir[A1]=-100000.0, location[A1]=left, closeToBar[A1], transHistory[robotAckAttention, A1], transHistory[robotGreeted, A1], transHistory[agentOrdered, A1], transHistory[robotServed, A1], transHistory[transComplete, A1], facePos[A1]=[660.9139072022265, 494.2966233427158, 561.7378637718438], ?seeksAttention[A1], !bodyLook[A1], request[A1]=coke, !faceLook[A1], !faceSeen[A1], bodyOri[A2]=-100000.0, !badASR[A2], visible[A2], faceDir[A2]=-100000.0, location[A2]=right, closeToBar[A2], transHistory[robotAckAttention, A2], transHistory[robotGreeted, A2], transHistory[agentOrdered, A2], transHistory[robotServed, A2], transHistory[transComplete, A2], facePos[A2]=[283.36932196608814, -426.9544626558899, -121.81771671730053], ?seeksAttention[A2], !bodyLook[A2], request[A2]=juice, !faceLook[A2], faceSeen[A2], lastEvent[]=outputDone]

-- 4/26/12 11:11:41:856 kinect-speech: 1: recognized: Auf Wiedersehen (0,101719476282597) -1,54182686551049 (0,317173146349584)

-- 4/26/12 11:11:42:080 state: 1: Relations: [inTrans[]=nil, bodyOri[A1]=-100000.0, !badASR[A1], visible[A1], faceDir[A1]=-100000.0, location[A1]=left, closeToBar[A1], transHistory[robotAckAttention, A1], transHistory[robotGreeted, A1], transHistory[agentOrdered, A1], transHistory[robotServed, A1], transHistory[transComplete, A1], facePos[A1]=[995.9308535433329, 254.21260272561935, 951.0358097907294], ?seeksAttention[A1], !bodyLook[A1], request[A1]=coke, !faceLook[A1], !faceSeen[A1], bodyOri[A2]=-100000.0, !badASR[A2], visible[A2], faceDir[A2]=-100000.0, location[A2]=right, closeToBar[A2], transHistory[robotAckAttention, A2], transHistory[robotGreeted, A2], transHistory[agentOrdered, A2], transHistory[robotServed, A2], transHistory[transComplete, A2], facePos[A2]=[294.21016438791344, -430.9248132569284, -161.52224767191774], ?seeksAttention[A2], !bodyLook[A2], request[A2]=juice, !faceLook[A2], faceSeen[A2], lastEvent[]=outputDone]

# References

Foster, M. E., Gaschler, A., & Giuliani, M. (2013). How can I help you? Comparing engagement classification strategies for a robot bartender. In *Proceedings of the ACM International Conference on Multimodal Interaction (ICMI 2013)* (pp. 255–262). Sydney, Australia: ACM Press. http://doi.org/10.1145/2522848.2522879

Foster, M. E., Gaschler, A., Giuliani, M., Isard, A., Pateraki, M., & Petrick, R. P. A. (2012). Two people walk into a bar: dynamic multi-party social interaction with a robot agent. In *Proceedings of the 14th ACM International Conference on Multimodal Interaction (ICMI 2012)*. Santa Monica, USA: ACM Press. http://doi.org/10.1145/2388676.2388680
